# Mechanisms Used for Genomic Proliferation by Thermophilic Group II Introns

**DOI:** 10.1371/journal.pbio.1000391

**Published:** 2010-06-08

**Authors:** Georg Mohr, Eman Ghanem, Alan M. Lambowitz

**Affiliations:** 1 Institute for Cellular and Molecular Biology, University of Texas at Austin, Austin, Texas, United States of America; 2 Department of Chemistry and Biochemistry, University of Texas at Austin, Austin, Texas, United States of America; 3 Section of Molecular Genetics and Microbiology, School of Biological Sciences, University of Texas at Austin, Austin, Texas, United States of America; National Center for Biotechnology Information, United States of America

## Abstract

Mobile group II introns, which are found in bacterial and organellar genomes, are site-specific retroelments hypothesized to be evolutionary ancestors of spliceosomal introns and retrotransposons in higher organisms. Most bacteria, however, contain no more than one or a few group II introns, making it unclear how introns could have proliferated to higher copy numbers in eukaryotic genomes. An exception is the thermophilic cyanobacterium *Thermosynechococcus elongatus*, which contains 28 closely related copies of a group II intron, constituting ∼1.3% of the genome. Here, by using a combination of bioinformatics and mobility assays at different temperatures, we identified mechanisms that contribute to the proliferation of *T. elongatus* group II introns. These mechanisms include divergence of DNA target specificity to avoid target site saturation; adaptation of some intron-encoded reverse transcriptases to splice and mobilize multiple degenerate introns that do not encode reverse transcriptases, leading to a common splicing apparatus; and preferential insertion within other mobile introns or insertion elements, which provide new unoccupied sites in expanding non-essential DNA regions. Additionally, unlike mesophilic group II introns, the thermophilic *T. elongatus* introns rely on elevated temperatures to help promote DNA strand separation, enabling access to a larger number of DNA target sites by base pairing of the intron RNA, with minimal constraint from the reverse transcriptase. Our results provide insight into group II intron proliferation mechanisms and show that higher temperatures, which are thought to have prevailed on Earth during the emergence of eukaryotes, favor intron proliferation by increasing the accessibility of DNA target sites. We also identify actively mobile thermophilic introns, which may be useful for structural studies, gene targeting in thermophiles, and as a source of thermostable reverse transcriptases.

## Abstract 2

Studies of mobile group II introns from a thermophilic cyanobacterium reveal how these introns proliferate within genomes and might explain the origin of introns and retroelements in higher organisms.

## Author Summary

Group II introns are bacterial mobile elements thought to be ancestors of introns and retroelements in higher organisms. They comprise a catalytically active intron RNA and an intron-encoded reverse transcriptase, which promotes splicing of the intron from precursor RNA and integration of the excised intron into new genomic sites. While most bacteria have small numbers of group II introns, in the thermophilic cyanobacterium *Thermosynechococcus elongatus*, a single intron has proliferated and constitutes 1.3% of the genome. Here, we investigated how the *T. elongatus* introns proliferated to such high copy numbers. We found divergence of DNA target specificity, evolution of reverse transcriptases that splice and mobilize multiple degenerate introns, and preferential insertion into other mobile introns or insertion elements, which provide new integration sites in non-essential regions of the genome. Further, unlike mesophilic group II introns, the thermophilic *T. elongatus* introns rely on higher temperatures to help promote DNA strand separation, facilitating access to DNA target sites. We speculate how these mechanisms, including elevated temperature, might have contributed to intron proliferation in early eukaryotes. We also identify actively mobile thermophilic introns, which may be useful for structural studies and biotechnological applications.

## Introduction

Mobile group II introns are bacterial and organellar retrotransposons that are hypothesized to be ancestors or closely related to ancestors of spliceosomal introns and retrotransposons in higher organisms (reviewed in [1],[2]). They consist of a catalytically active intron RNA (“ribozyme”) and an intron-encoded protein (IEP), which has reverse transcriptase (RT) activity. Group II intron RNAs typically show little sequence conservation but have conserved secondary and tertiary structures that consist of six interacting double-helical domains (DI–DVI) [3],[4]. The folded RNA catalyzes its own splicing via two sequential transesterification reactions that are the same as those for spliceosomal introns in higher organisms and yield spliced exons and an excised intron lariat RNA [5]. For mobile group II introns, the IEP assists splicing by stabilizing the catalytically active RNA structure (“maturase activity”) and then remains bound to the excised intron lariat RNA in a RNP (ribonucleoprotein particle) [6]–[8]. The latter promotes intron mobility by a mechanism that involves reverse splicing of the intron RNA directly into a DNA strand, reverse transcription of the inserted intron RNA by the IEP, and integration of the resulting intron cDNA into the genome by host enzymes [9]–[13]. This mechanism is used by group II introns both to retrohome into specific DNA target sites at high frequency and to retrotranspose into ectopic sites that resemble the retrohoming site at low frequency, and ancestral mobile group II introns may have used the same mechanism to invade and proliferate within the nuclear genomes of early eukaryotes, before evolving into spliceosomal introns, snRNAs, and non-LTR-retrotransposons [1],[2].

Group II introns are common in eubacteria and in the mitochondrial (mt) and chloroplast (cp) genomes of fungi and plants but are rare in archaea, with the few known examples of archaeal group II introns attributed to horizontal transfer from eubacteria [14],[15]. This phylogenetic distribution is consistent with a scenario in which mobile group II introns evolved in eubacteria and were transferred to eukaryotes with bacterial endosymbionts that gave rise to eukaryotic organelles [16],[17]. Bacteria contain eight lineages of mobile group II introns, which are distinguished by different IEP types (bacterial A–F, mitochondrial-like (ML) and chloroplast-like (CL)) and distinct RNA structural subgroups (IIA (ML), IIB (CL and bacterial lineages A, B, and D–F), and IIC (bacterial lineage C)) [18]. Notably, while all eight group II intron lineages are found in bacteria with extensive horizontal transfer between different species [14],[19], only the ML and CL lineages are also found in organelles, consistent with the possibility that they were associated with the bacterial endosymbionts that evolved into mitochondria and chloroplasts.

Evolutionary scenarios for the evolution of group II introns into spliceosomal introns suggest that group II introns harbored by bacterial endosymbionts invaded the host's nuclear genome, where they proliferated and degenerated, with the group II intron RNA domains evolving into spliceosomal snRNAs that form the core of a common splicing apparatus for multiple dispersed introns [2],[20]. Key aspects of this scenario are supported by experimental evidence, including structural and functional similarities between group II intron domains and snRNAs and numerous examples of group II introns that are fragmented into two or three segments that functionally reassociate to catalyze *trans*-splicing [1],[2],[21],[22]. Most bacteria, however, contain no more than one or a few group II introns [23], suggesting that group II intron mobility is tightly controlled and/or that mutations that lead to uncontrolled intron proliferation are lost rapidly by purifying selection. Thus, it is unclear how group II introns could have proliferated to higher copy numbers in nuclear genomes.

Although group II intron proliferation is rare in bacteria, it is evident in smaller organellar genomes, the most striking example being *Euglena* cp DNA, which contains ∼150 group II introns [24]. Most of the *Euglena* introns are highly degenerate, lacking different domains, with some (referred to as group III introns) containing only DI-like and DVI-like structures and even lacking DV, which is catalytically essential. These degenerate introns presumably rely on protein factors and/or *trans*-acting RNAs to promote RNA splicing. Only two of the *Euglena* cp group II introns encode RTs, which may act in *trans* to promote splicing and mobility of the ORF-less introns [25]. Although the *Euglena* group II introns can potentially provide insight into mechanisms involved in intron proliferation and generation of a common splicing apparatus, they have been largely intractable to detailed analysis.

Another instance of group II intron proliferation has been revealed by genomic sequencing of the thermophilic cyanobacterium *Thermosynechococcus elongatus* strain BP-1, which contains 28 group II introns comprising ∼1.3% of the genome [26]. These *T. elongatus* group II introns are closely related to each other and appear to have proliferated from a single group II intron. Here, we used bioinformatic analysis and intron mobility assays at different temperatures to identify four mechanisms that contribute to the proliferation of the *T. elongatus* introns. Our results provide insight into how group II introns proliferate within genomes; show how higher temperatures, which are thought to have prevailed on Earth during the emergence of eukaryotes, can contribute to this process; and identify actively mobile thermophilic group II introns, which may be useful for structural studies and biotechnological applications.

## Results

### Characteristics of *T. elongatus* Group II Introns

**Figure 1:**
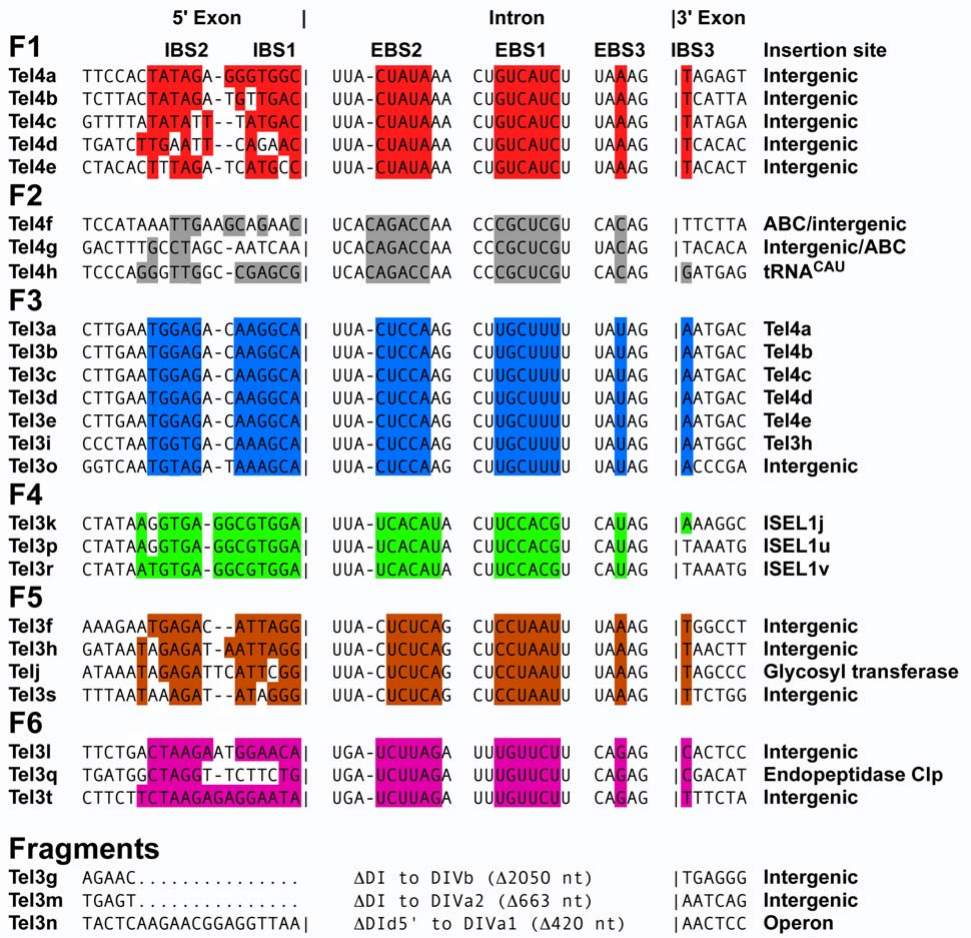
*T. elongatus* group II Intron families and insertion sites. The 25 intact introns are classified into six families (F1–F6) based on their EBS sequences. Three other introns are fragments (TeI3g retains ∼340 nts of the 3′ part of the intron starting in the En domain of the IEP; TeI3m lacks regions upstream of DIVa(3′); and TeI3n has a large internal deletion between DId(5′) and DIVa1). Colors highlight EBS sequences and complementary nucleotide residues in the IBS sequences. The EBS2 sequence of TeI4h could not be identified unambiguously from the secondary structure model and was defined by in vivo selections with donor and recipient plasmids in which potential EBS2 and IBS2 nucleotide residues were randomized (G.M. and A.M.L., unpublished data).

Figure 1 lists the *T. elongatus* introns classified according to intron family (F1–F6) by criteria described below, along with their insertion sites in the *T. elongatus* genome. All 28 introns are closely related to each other (85%–100% sequence identity in pairwise comparisons). Twenty-five of the *T. elongatus* introns are intact containing all six conserved RNA domains, but three are fragments that have undergone large deletions (TeI3g, TeI3m, and TeI3n; Figure 1). Eight of the intact introns (TeI4a–h) contain ORFs encoding IEPs (see below), while the remaining 17 introns (TeI3a–t) lack ORFs but are otherwise closely related to the ORF-containing introns. The closest known relative of the *T. elongatus* introns is EcI5, a previously characterized *Escherichia coli* CL/IIB1 intron (∼50% sequence identity in pairwise comparisons [27],[28]).

**Figure 2:**
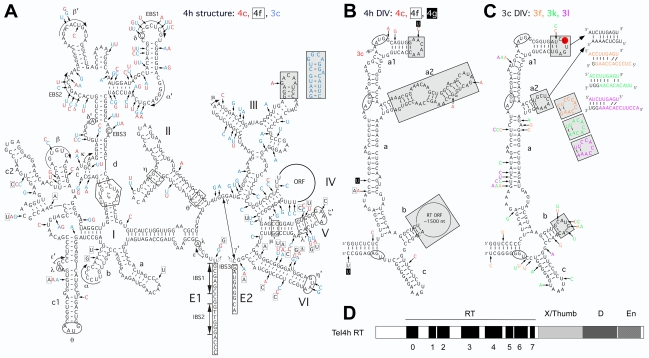
*T. elongatus* group II intron RNA secondary structure and IEP. (A) Predicted secondary structure of TeI4h. Differences in TeI4c, TeI4f, and TeI3c are indicated in red, boxed, and blue letters, respectively. The structure consists of six conserved domains (DI–DVI). Subdomains and further subdivisions are denoted with letters followed by numbers (e.g., DIc1). Greek letters indicate nucleotide sequences involved in long-range tertiary interactions [2]; 5′ and 3′ exon (E1 and E2, respectively) are boxed; and splice sites are indicated by open arrowheads. The gray boxes show a region of DIII that is replaced by a different sequence in TeI3c (blue, inset). (B) Secondary structure of DIV of ORF-containing TeI4 introns. The figure shows the secondary structure of DIV of TeI4h, with differences in TeI4c, 4f, and 4g indicated in red, boxed, and white letters in black boxes, respectively. The two potential start codons and the stop codon of the intron ORF are circled, and the arrow between the two potential start codons indicates the site at which TeI3c and other F3 introns insert into TeI4c and other F1 introns, resulting in the formation of twintrons. Regions that differ substantially in the ORF-less TeI3 introns are shaded gray. (C) Secondary structure of DIV of the ORF-less TeI3 introns. The figure shows the secondary structure of TeI3c, with differences in TeI3f, 3k, and 3l indicated in orange, green, and purple, respectively. Regions that differ from the ORF-containing TeI4 introns are shaded gray. Potential base pairings between the DIVa1 and DIVa2 loops are indicated at the upper right. A red circle highlights the extra U residue in DIVa1 of ORF-less introns (see also Figure S2). (D) Schematic of the TeI4h IEP. Conserved protein domains are: RT, containing conserved amino acid sequence blocks RT1–7 characteristic of the finger and palm regions of retroviral RTs; X/Thumb, region associated with maturase activity and corresponding in part to the RT thumb; D, DNA binding; and En, DNA endonuclease; RT-0 is a region conserved in the RTs of non-LTR retroelements [1],[29]. Multiple sequence alignments of the TeI4h and other IEPs are shown in Figure S1.

Except for the presence or absence of the intron ORF, the 25 intact *T. elongatus* introns have very similar predicted RNA secondary structures, which are characteristic of subclass IIB1 introns and closely resemble that of EcI5 [28]. Figure 2A–C show a secondary structure model for TeI4h, one of the introns studied in detail below, and key differences in representatives of other *T. elongatus* intron families. As for other group II introns, the predicted structure consists of the six conserved RNA domains (DI–DVI) with a series of conserved motifs (denoted by Greek letters, EBS (exon-binding site), and IBS (intron-binding site)). The latter are involved in a series of long-range interactions that help fold the intron RNA into the catalytically active tertiary structure. Notable regions are DI and DV, which together comprise the minimal catalytic core; DIV, which encodes the IEP in the loop of subdomain DIVb; and DVI, which contains the branch-point A-residue [2]. Many of the sequence differences between the *T. elongatus* introns correspond to reciprocal changes in stem regions, but some introns have deviations in conserved structures or motifs that are expected to impair ribozyme activity (e.g., TeI4f and TeI4g have mispairings in the upper stem of DV and the lower stem of DVI). Inactive group II introns that have lost mobility functions are common in bacteria and organelles and presumably reflect selective pressure against intron mobility, which is deleterious to the host [1].

### Characteristics of the *T. elongatus* IEPs

TeI4a–h have long ORFs in DIV that encode predicted IEPs of 561 amino acid residues with RT, X/thumb, DNA binding (D), and DNA endonuclease (En) domains homologous to those of other group II IEPs (Figure 2D) [1],[2],[29]. Seven of the ORFs show strong conservation of amino acid sequences residues known to be required for activity of other group II IEPs (see alignments in Figure S1), but one has an early frameshift as well as multiple premature stop codons and other sequence deviations (TeI4d, not shown in the alignment). Like the intron RNAs, the IEPs are closely related to each other (84%–100% identity in pairwise comparisons, excluding TeI4d), and their closest known relative is the EcI5 IEP (53%–55% identity in pairwise comparisons; Figure S1).

Two introns (TeI4f and 4h) have continuous ORFs, while in five other introns (TeI4a, b, c, d, and e) the ORF is interrupted by insertion of an ORF-less intron (TeI3a, b, c, d, and e, respectively) between the first two ATGs, which are separated by only five codons (Figure 2B, Figure S2). This configuration in which one intron inserts into another is known as a “twintron” [24]. The *T. elongatus* twintrons could potentially encode active IEPs initiated either from the first AUG after splicing of the inner intron or from the second AUG without splicing of the inner intron. In the remaining intron, TeI4g, the ORF is interrupted by an insertion element (TeI4g::ISEL2f) [26]. Thus, excluding TeI4d and TeI4g, six of the *T. elongatus* introns (TeI4a, b, c, e, f, and h) could potentially encode active IEPs.

### The ORF-Less *T. elongatus* Introns Were Derived from a Single ORF-Containing Intron by ORF Deletion

**Figure 3:**
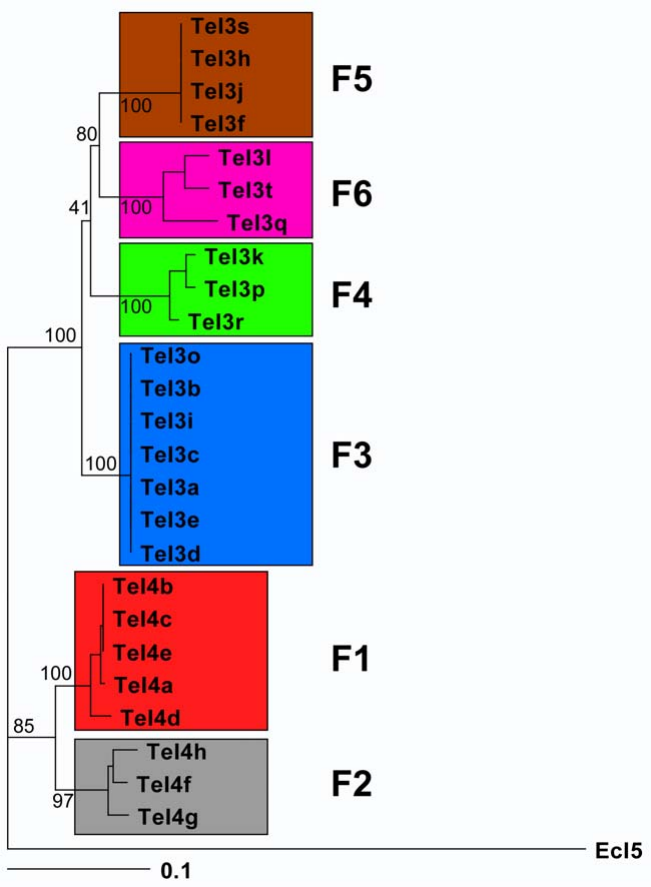
Phylogeny of *T. elongatus* introns. The figure shows a phylogram for all 25 intact *T. elongatus* introns. TeI4 introns were aligned with TeI3 introns by deleting ORF sequences in DIVb (positions 755–2290 of TeI4h). RNA sequences were aligned with ClustalX [49], and the alignment was refined manually and used as input for Phylip (ver. 3.69, with default parameters [50]). The phylogenies were generated with program modules DNAdist and DNAcomp using all of the Distance settings (F84, Kimura, Jukes-Cantor, LogDet) independently and varying the out-group (EcI5 or random Te intron). Trees were visualized with Treeview [51],[52] and were essentially the same regardless of distance or out-group settings. Support for the major groupings of the phylogram was obtained by bootstrapping 1,000 data sets (using Seqboot from Phylip ver. 3.69) and using these as input for DNAdist. The output of the latter program was then used to obtain a consensus tree with Consense. The numbers indicate the percentage of times a particular grouping occurred in the 1,000 data sets.

All 17 ORF-less *T. elongatus* introns (TeI3a–t) have a ∼1.5 kb deletion in subdomain DIVb, which removes most of the ORF (Figure 2B and C). Sequence alignments show that the break-points of this deletion are the same in all 17 introns and that remnants of the N- and C-termini of the ORF are clearly discernible on either side (Figure S2). All of the ORF-less introns also have a single extra U residue in DIVa1, which disrupts the remnant reading frame, and a second smaller deletion in DIVa2 (Figure 2B and C; Figure S2). These findings most simply suggest that the 17 ORF-less introns arose from a single ancestral intron that underwent the deletion and then proliferated. Phylogenetic analysis based on alignments of intron sequences supports this hypothesis and indicates that the ORF deletion was an early event that occurred before the divergence of the *T. elongatus* introns into different families (Figures 3 and S3, and see below). Notably, in the ORF-less introns, the terminal loop of DIVa2 resulting from the smaller deletion in DIV has co-varied with the terminal loop of DIVa1 in a manner suggesting a base-pairing interaction between the loops (Figure 2C). As DIVa is a critical binding region for the IEP in subgroup IIA introns [30], these changes could be pertinent to IEP recognition.

### The *T. elongatus* Introns Have Diverged into Six Families with Different Target Sites

Group II introns recognize DNA target sequences by using both the IEP and base pairing of the intron RNA [31],[32]. In group IIB introns, the base-pairing interactions involve intron RNA sequences denoted EBS1, 2, and 3 and complementary DNA target sequences denoted IBS1 and 2 in the 5′ exon and IBS3 in the 3′ exon [28],[33],[34]. We noticed that the *T. elongatus* introns could be divided into six families, F1–F6, based upon their EBS1, 2 and 3 sequences (Figure 1), and phylogenetic analysis indicated that each of these families corresponds to a distinct clade (Figures 3 and S3). Most of the introns are inserted at genomic sites with largely complementary IBS sequences (one or no mismatches), suggesting insertion by retrohoming, but some are inserted at sites with more poorly matched IBS sequences, suggesting insertion by infrequent retrotranspositions. Because the EBS/IBS interactions in the precursor RNA are required for RNA splicing, only those introns inserted by retrohoming at sites with complementary IBS sequences are expected to splice efficiently. Most introns with poorly matched IBS sequences and some introns with well-matched IBS sequences are inserted at sites in intergenic regions, where their splicing ability is less likely to affect host gene expression.

The eight ORF-containing introns are divided into two families (F1 and F2), while the 17 ORF-less introns are divided into four families (F3–6; Figure 1). The F1 introns (TeI4a–e) are inserted in intergenic regions, as are two of the F2 introns (TeI4f and 4g). Notably, the 5′ exon of TeI4f and the 3′ exon of TeI4g correspond to 5′ and 3′ segments of an ABC transporter pseudo-gene, respectively, suggesting these introns were derived from an exon-shuffling homologous recombination event between two ancestral introns. The remaining F2 intron, TeI4h, is inserted in a gene encoding the single copy of tRNA_Ile_^CAU^, which is putatively essential [26]. The site of insertion in the tRNA gene is one with good EBS/IBS pairings, suggesting that TeI4h inserted by retrohoming and can splice to produce a functional tRNA.

F3 consists of seven identical ORF-less introns, five of which (TeI3a–e) are inserted at a conserved site between the two closely spaced start codons of the RT ORF of F1 introns, while TeI3i is inserted at the same site in the remnant ORF of TeI3h, and TeI3o is inserted in an intergenic region. F4 consists of three introns inserted at a conserved site in insertion element ISEL1, a member of the IS200 insertion element family [26]. F5 consists of three introns inserted in intergenic regions and a fourth intron (TeI3j) inserted within a glycosyl transferase gene. Finally, F6 consists of two introns inserted in intergenic regions and one intron inserted near the 3′ end of gene t112453. The finding that only a small number of *T. elongatus* introns are inserted within genes may reflect purifying selection against insertions that are deleterious to the host, but mechanisms for actively avoiding insertion within genes are also possible.

Importantly, most of the ORF-less introns are inserted at sites with good EBS/IBS pairings, suggesting insertion via retrohoming, further evidence that the ORF-less introns are actively mobile (Figure 1). The larger number of ORF-less introns presumably reflects that they are more efficiently mobile than ORF-containing introns due to their smaller size and more compact structure. The latter makes them less susceptible to degradation by host nucleases, which appears to be a major means of controlling group II intron mobility in bacteria [35].

### Derivatives of the TeI4h Intron Are Mobile and Thermophilic

**Figure 4:**
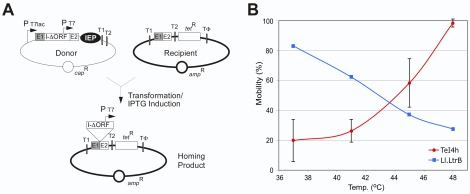
TeI4h intron mobility assays. (A) *E. coli* genetic assay of intron mobility. The Cap^R^ donor plasmid uses a T7lac promoter (P_T7lac_) to express a ΔORF intron (I-ΔORF) with short flanking 5′ and 3′ exons (E1 and E2, respectively) and the IEP downstream of E2. The intron, which carries a T7 promoter (P_T7_) in DIVb, integrates into a target site (ligated E1–E2 sequences) cloned in an Amp^R^ recipient plasmid upstream of a promoterless *tet^R^* gene, thereby activating that gene. The donor and recipient plasmids are derivatives of pACD2X and pBRR-tet, respectively (see Materials and Methods). The assays are done in *E. coli* HMS174(DE3), which contains an IPTG-inducible T7 RNA polymerase, with intron expression induced with 500 µM IPTG for 1 h at different temperatures. Mobility efficiencies are calculated as the ratio of (Tet^R^+Amp^R^)/Amp^R^ colonies. (B) Mobility efficiency of the TeI4h-ΔORF (blue) and Ll.LtrB-ΔORF (red) introns as a function of induction temperature. The donor plasmid for the Ll.LtrB-ΔORF intron was pACD2X [47].

To directly assay mobility of the *T. elongatus* introns, we used an *E. coli* plasmid assay in which an intron with a phage T7 promoter inserted near its 3′ end is expressed from a donor plasmid and integrates into a target site cloned in a recipient plasmid upstream of a promoterless tetracycline-resistance (*tet^R^*) gene, thereby activating that gene (Figure 4A; [36],[37]). Because the intron is expressed from a donor plasmid, the IEP must splice the intron RNA to generate RNPs, which then promote integration of the intron into the DNA target site. Previous studies showed that the *Lactococcus lactis* Ll.LtrB intron is efficiently mobile in this *E. coli* assay, reflecting that it has a wide host range and is not dependent upon host-specific factors for RNA splicing or intron mobility [13],[36],[38], and we anticipated this would also be the case for the *T. elongatus* introns.

For the mobility assays with the *T. elongatus* introns, the Cap^R^ (chloramphenicol-resistant) intron-donor plasmid uses a T7lac promoter (P_T7lac_) to express a precursor RNA containing a ΔORF-derivative of the intron RNA (I-ΔORF) with short flanking exons and a phage T7 promoter (P_T7_) inserted in place of the intron ORF in DIVb. The IEP, which splices and mobilizes the intron, is expressed from a position downstream of the 3′ exon (E2). This configuration using an intron RNA with the ORF deleted and the IEP expressed from downstream of E2 gives high mobility frequencies for other group II introns [28],[36] and facilitates the mixing and matching of intron RNAs and IEPs in experiments below. The Amp^R^ (ampicillin-resistant) recipient plasmid contains the intron target site (i.e., ligated-exon sequences flanking the intron-insertion site) cloned upstream of a promoterless *tet* ^R^ gene. For mobility assays, the donor and recipient plasmids are co-transformed into *E. coli* HMS174(DE3), which contains an IPTG (isopropyl β-D-1-thiogalactopyranoside)-inducible T7 RNA polymerase. After induction of donor plasmid expression with IPTG, cells are plated on Luria-Bertani (LB) medium containing tetracycline plus ampicillin or amplicillin alone, and mobility efficiencies are calculated as the ratio of (Tet^R^+Amp^R^)/Amp^R^ colonies.

We focused first on TeI4h, which is inserted within the tRNA_Ile_^CAU^ gene because it contains a continuous ORF and is inserted within an essential gene at a site with good EBS/IBS pairings. These characteristics imply insertion via retrohoming and active splicing to produce a functional tRNA. Nevertheless, TeI4h has features that deviate from the canonical group II intron structure, including a 5′ T residue and a mispairing in the δ-δ′ interaction in the intron RNA (Figure 2A) and YAGD instead of the highly conserved YADD motif at the RT active site in the IEP (Figure S1).

Table 1 summarizes the mobility efficiencies for donor plasmids expressing different derivatives of the TeI4h-ΔORF intron and IEP at different induction temperatures. At 37°C, the wild-type TeI4h-ΔORF intron and IEP combination had very low mobility efficiency (2.5×10^−5^%), but changing the IEP's YAGD sequence to YADD increased the mobility efficiency dramatically to 3.4%. This modified IEP, denoted IEP-4h*, was used in all subsequent constructs. Combining the TeI4h* IEP with a modified intron (denoted TeI4h*), which has the change C326T in δ′ to restore the δ-δ′ pairing, increased the mobility efficiency further to 39%. Surprisingly, however, combining the TeI4h* IEP with a modified intron in which the 5′-nucleotide residue was changed from T to the highly conserved 5′ G residue found in other group II introns decreased the mobility efficiency to 0.5%, and combining all three changes gave a mobility efficiency of only 8.4%. Thus surprisingly, the unusual 5′ T-residue is favored in TeI4h. Sequencing of retrohoming products from this experiment and from target-site definition experiments described below showed that the inserted TeI4h* intron begins with the unusual 5′ T-residue in all cases (>600 sequences), confirming use of a corresponding 5′ U-residue in the intron RNA for both RNA splicing and reverse splicing. The maximally efficient donor plasmid expressing the TeI4h*-ΔORF intron with the C236T mutation restoring δ-δ′ and the TeI4h* IEP, with the mutation changing YAGD to YADD at the RT active site, is designated pACD2-TeI4h*/4h* to denote the intron RNA/IEP combination.

**Table 1:** Mobility efficiencies of TeI4h and effect of mutations at different temperatures.

			Mobility (%)		
Intron	IEP		Temp (°C)		
		37	41	45	48
4h	4h	2.5×10^−5^	N.D.	N.D.	6.7×10^−2^
4h	4h^*^	3.4	2.4	34	100
4h^C236T^ (4h^*^)	4h^*^	39	37	81	100
4h^T1G^	4h^*^	0.5	1.2	11	60
4h^T1G/C236T^	4h^*^	8.4	27	82	100

Mobility assays were done in *E. coli*, as described in Figure 4 and Materials and Methods, using different TeI4h/4h donor plasmids that express the wild-type or mutant TeI4h-ΔORF introns and IEPs, and a recipient plasmid that contains the TeI4h target site (i.e., ligated E1–E2 sequences). Donor plasmid expression was induced with 500 µM IPTG for 1 h at different temperatures. Mobility efficiencies were calculated as the ratio of (Tet^R^+Amp^R^)/Amp^R^ colonies. 4h* IEP, YAGD changed to YADD; C236T (TeI4h*), intron position 236 (δ) changed from C to T; T1G, intron position 1 changed from T to G. N.D., not determined.

At higher temperatures, the mobility efficiency of TeI4h and its derivatives increased dramatically, with the mobility efficiency of the optimal TeI4h*/4h* construct as well as several of the suboptimal constructs reaching 100% at 48°C (Table 1 and Figure 4B). By contrast, the mobility efficiency of the mesophilic Ll.LtrB-ΔORF intron expressed from an analogous donor plasmid decreased with increasing induction temperature, with the residual ∼20% mobility at 48°C likely reflecting integrations that occurred at 37°C prior to induction or after plating. The native TeI4h/4h construct, which expresses the wild-type ΔORF intron and IEP without modification of the δ-δ′ pairing or YAGD sequence, had low mobility efficiency even at 48°C (6.7×10^−2^%), indicating that one or both of these suboptimal features inhibits mobility regardless of temperature (Table 1). We note that the mobility of the TeI4h at 48°C presumably relies either on residual activity of T7 RNA polymerase at the higher temperature and/or on RNPs made at 37°C prior to the temperature shift. Together, the above findings show that the TeI4h-ΔORF intron and IEP are thermophilic, presumably reflecting adaptation for retromobility in the native host *T. elongatus*.

### Mobility Assays with Other *T. elongatus* Introns

The finding that the *T. elongatus* ORF-less introns likely evolved from a single intron by ORF deletion and then proliferated to new sites by retrohoming suggested that their splicing and mobility might be promoted by one or more of the IEPs encoded by other *T. elongatus* introns. To test this hypothesis, we carried out *E. coli* mobility assays at 48°C with donor plasmids expressing different combinations of *T. elongatus* introns and IEPs (Table 2). The intron RNAs tested included at least one representative of each intron family (the ORF-less introns TeI3c, 3k, 3f, and 3l, all of which have good EBS/IBS pairings with their flanking exons, and TeI4h*-ΔORF, 4c-ΔORF, and 4f-ΔORF, with ORF deletions matching the deletion break points in the naturally ORF-less introns; see Materials and Methods). The IEPs tested were TeI4h*, 4a, 4b, 4c, 4e, 4f, and 4g, the latter with the IS element precisely deleted. The TeI4c ORF, which in *T. elongatus* is present in a twintron with TeI3c inserted between the first two ATGs, was expressed from either ATG. After this choice of ATGs was found to have little effect on mobility efficiency (see below), the remaining twintron IEPs were expressed only from the second ATG. The recipient plasmids contained the natural target site (i.e., ligated-exon sequences) for each intron (Figure 1; see Materials and Methods).

**Table 2:** Mobility efficiencies for intron donor plasmids expressing different combinations of *T. elongatus* group II intron RNAs and IEPs.

					Mobility (%)				
Intron	Family				IEP				
		4h	4a (2^nd^)	4b (2nd)	4c (1st)	4c (2nd)	4e (2nd)	4f	4g
4h	F2	100	1.8×10^−3^	5.6×10^−4^	5.4×10^−3^	3.0×10^−3^	1.8×10^−3^	9.0×10^−3^	3.7×10^−2^
4c	F1	1.4×10^−4^	3.6×10^−2^	1.2×10^−2^	2.6×10^−2^	2.3×10^−2^	2.5×10^−2^	1.1×10^−2^	1.1×10^−2^
4f	F2	0	N.D.	N.D.	0	0	N.D.	2.4×10^−4^	N.D.
3c	F3	6.4×10^−2^	0.62	3.8	6.7	7.7	0.32	2.0×10^−3^	2.0×10^−2^
3k	F4	3.7×10^−3^	1.9×10^−4^	3.5×10^−4^	3.0×10^−2^	5.9×10^−3^	2.1×10^−4^	N.D.	2.1×10^−4^
3f	F5	1.9×10^−3^	5.6×10^−3^	6.9×10^−3^	2.4×10^−3^	4.4×10^−4^	9.2×10^−3^	N.D.	2.7×10^−4^
3l	F6	2.6×10^−3^	1.8×10^−2^	0.50	2.4	0.96	4.6×10^−2^	N.D.	4.5×10^−4^

Mobility assays were done in *E. coli*, as described in Figure 4 and Materials and Methods, using a donor plasmid that expresses the indicated TeI3 or TeI4-ΔORF intron and IEP combination and a recipient plasmid that contains the target site for the intron (i.e., ligated E1–E2 sequences). Donor plasmid expression was induced with 500 µM IPTG for 1 h at 48°C, and mobility efficiencies were calculated as the ratio of (Tet^R^+Amp^R^)/Amp^R^ colonies. PCR and sequencing of 3 to 10 Tet^R^+Amp^R^ colonies confirmed insertion of the intron at the target in all cases, except for TeI3f with the 4a, b, e, and g IEPs, where the low mobility efficiency calculated from ratios of antibiotic-resistant colonies thus represent an upper limit. F1–F6 denote intron families. 1st and 2nd denote the initiation codon used for expression of twintron IEPs, where the intron ORF has been interrupted by the insertion of another group II intron between the first two ATGs. The TeI4f-ΔORF intron was tested with modifications that improved the EBS/IBS pairings (see Materials and Methods), and the TeI4g IEP was tested with the inserted IS element precisely deleted based on the alignment in Figure S1. Similar mobility efficiencies (less than 5-fold difference) were obtained in one or two repeats of the mobility assays in all cases. N.D., not determined.

The results, summarized in Table 2, show that the TeI4h* IEP has very high specificity for its cognate intron. Its mobility efficiency with the TeI4h* RNA was 100%, while its mobility efficiencies with all other intron RNAs were 10^4^- to 10^6^-fold lower (TeI4c, 3c, 3k, 3f, and 3l; 6.4×10^−2^ to 1.4×10^−4^%). The other IEPs were less specific. Among these, the TeI4c IEP was the most active. Expressed from either the first or second ATG, it mobilized the TeI4c-ΔORF RNA with efficiencies of 2.6 and 2.3×10^−2^%, respectively, while it mobilized representatives of other intron families at comparable (4.4×10^−4^ to 3×10^−2^%; 4h, 3k, 3f) or higher efficiencies (0.96%–7.7%; 3c and 3l). We note that low mobility efficiencies for some introns are a consequence of suboptimal natural target sites, as the TeI4c IEP could also promote mobility of either TeI4c-ΔORF or TeI3c at 60%–80% efficiency with other target sites (Table S1 and unpublished data). The TeI4a, b, c, and e IEPs showed different degrees of activity and specificity for different introns, but each had the ability to splice and mobilize multiple introns to some degree. Among the naturally ORF-less introns, TeI3c is most active with the widest variety of IEPs; TeI3l has relatively high activity (0.5%–2.4%) with a subset of these IEPs; and TeI3f and TeI3k have low activity with most of the IEPs. The TeI4f IEP and restored TeI4g IEP with the IS element deleted gave low mobility efficiencies (3.7×10^−2^ to 2.1×10^−4^%) with all introns tested, and the TeI4f RNA showed little or no mobility with any IEP tested, likely reflecting mutations that inhibit ribozyme activity, which is required for RNA splicing and reverse splicing (see above; Figure 2B). Together, the findings in this section indicate that some but not all of the *T. elongatus* IEPs have decreased specificity for their cognate intron, enabling them to mobilize multiple ORF-less introns as or more efficiently than the intron that encodes them.

### DNA Target Site Recognition

Another mechanism that could potentially contribute to proliferation of the *T. elongatus* introns is relaxed DNA target specificity. For previously characterized group II introns, the IEP recognizes sequences in the distal 5′-exon and 3′-exon regions of the DNA target and promotes DNA melting, enabling the intron RNA's EBS sequences to base pair to the target site's IBS sequences [31]. The EcI5 IEP, which is closely related to the *T. elongatus* IEPs, recognizes five different nucleotide residues flanking the IBS sequences, C–18, C–17, A–15, and A–14 in the distal 5′-exon region and T+5 in the 3′ exon [28]. A more relaxed DNA target specificity of the *T. elongatus* IEPs would enable intron RNAs with the same EBS sequences to insert at a greater number of sites.

**Figure 5:**
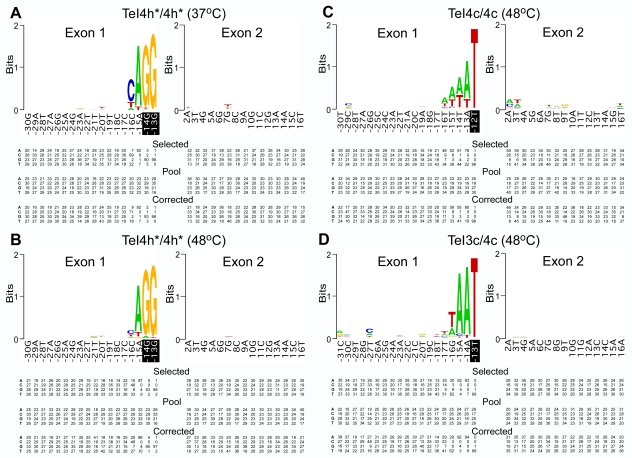
Identification of critical nucleotide residues in the distal 5′-exon and 3′-exon regions of the DNA target sites of *T. elongatus* introns. (A) Intron donor plasmid TeI4h*/4h* at 37°C. Intron donor plasmid TeI4h*/4h* at 48°C. (C) Intron donor plasmid TeI4c/4c at 48°C. (D) Intron donor plasmid TeI3c/4c at 48°C. Selection experiments were done in *E. coli* HMS174(DE3) containing the indicated intron donor plasmid and a recipient plasmid library randomized at the positions shown, as described in Materials and Methods. After selection by plating on LB medium containing antibiotics, Amp^R^+Tet^R^ colonies were analyzed by colony PCR and sequencing of the 5′- and 3′-integration junctions to identify nucleotide residues in active target sites. The WebLogo representation [39] depicts nucleotide frequencies at each randomized position in ∼100 selected target sites, corrected for biases in the initial pool based on sequences of ∼100 randomly chosen recipient plasmids [28]. The sequence of the intron-insertion site in the *T. elongatus* genome is shown, with white bases on black background indicating randomized nucleotides belonging to IBS2. Summarized below are nucleotide frequencies (percent) at each randomized position in (i) active target sites after intron insertion (“selected”), (ii) randomly chosen recipient plasmids from the original pool (“pool”), and (iii) active target sites corrected for nucleotide frequency biases in the initial pools (“corrected”). The latter were used to generate the WebLogos. In some cases, percentage totals do not equal 100 due to rounding off.

To identify nucleotide residues in the DNA target site that are recognized by the *T. elongatus* IEPs, we carried out selection experiments using the donor plasmids TeI4h*/4h*, TeI4c/4c, and TeI3c/4c with recipient plasmids in which the distal 5′-exon and 3′-exon regions potentially recognized by the IEP were randomized [28]. After selection for Tet^R^+Amp^R^ colonies in which the intron had inserted into the recipient plasmid, the 5′- and 3′-integration junctions in active target sites were amplified by colony PCR and sequenced. Figure 5 summarizes nucleotide frequencies at the randomized positions in active target sites in WebLogo format [39]. In each case, the nucleotide frequencies are based on sequencing of ∼100 active target sites and were corrected for nucleotide frequency biases in the libraries by sequencing a similar number of unselected recipient plasmids from the initial pools (Figure 5). Because we were uncertain about the boundaries of IBS2, the randomized regions in the recipient plasmids extended 1–2 nucleotide residues into IBS2 (shown in black in Figure 5) to confirm selection for complementary nucleotide residues in EBS2.

Figure 5A and B show selections at different temperatures for the donor plasmid TeI4h*/4h* in which the TeI4h* IEP promotes mobility of the TeI4h*-ΔORF intron. At 37°C, we see selection for two nucleotide residues in the distal 5′-exon region, A–15 and C–16, along with G–14 and G–13, which are part of IBS2 recognized by base pairing of C-residues at the corresponding positions in EBS2 of the intron RNA. Although the TeI4h IEP contains an En domain, there was no selection for any 3′-exon nucleotide, which is required for En cleavage by other group II IEPs [31]. At 48°C, we see similarly strong selection for the two EBS2 residues but somewhat weaker selection for A–15 and strongly decreased selection for C–16 compared to that at 37°C. Mobility assays confirmed that a mutation at position −16 (C–16G) inhibits mobility to a much greater extent at 37°C than at 48°C in agreement with the selection data (Figure S4). These findings likely reflect that the recognition of C–16 by the IEP is more stringently required for DNA melting at 37°C than at higher temperatures, which by themselves promote DNA melting. The selected distal 5′-exon sequence A–15, C–16 matches that at the TeI4h insertion site in the *T. elongatus* genome (Figure 1).

Figure 5C shows a similar selection at 48°C for the donor plasmid TeI4c/4c, in which the TeI4c IEP mobilizes its cognate TeI4c-ΔORF intron RNA. Here, we see selection for A or T at positions −13 to −16, which diminishes with increasing distance from the last nucleotide of IBS2 (T–12), along with weak selection for A or T at positions +2 and +3. This pattern most likely reflects selection for less stable AT base pairs that would facilitate melting of this region rather than any specific base recognition, potentially an example of a mobile intron that recognizes its DNA target site entirely by base pairing. The insertion site for TeI4c in the *T. elongatus* genome also shows A/T residues extending from −13 to −17 upstream of IBS2 and at +2 and +3 downstream of IBS3 (Figure 1).

Finally, Figure 5D shows a selection at 48°C for the donor plasmid TeI3c/4c, in which the same TeI4c IEP mobilizes the ORF-less intron TeI3c. Surprisingly, we now see strong selection for A–14 and A–15 upstream of IBS2, with weaker selection for A or T at position −16, suggesting that the TeI4c IEP makes a greater contribution to DNA target site recognition when paired with the non-cognate TeI3c RNA than with its own cognate TeI4c-ΔORF RNA. The selected sequence again matches the genomic insertion site for TeI3c (Figure 1). The apparently altered DNA target specificity of the TeI4c IEP when paired with the TeI3c RNA may reflect that the non-cognate intron RNA induces a protein conformation that interacts differently with the DNA target site or that the different intron RNA/DNA base-pairing interactions lead to differences in DNA target site recognition by the IEP. Additionally, we cannot exclude that a small number of nucleotide residues upstream of IBS2 are recognized in some unknown way by the intron RNA rather than the IEP. In summary, the selection experiments show that all three *T. elongatus* introns tested recognize DNA target sites almost entirely by base pairing of the intron RNA, with the IEP making a much smaller contribution than for previously analyzed mesophilic group II introns, particularly at elevated temperatures that help promote DNA strand separation.

## Discussion

Here, we analyzed group II introns that have proliferated within the genome of the thermophilic cyanobacterium *T. elongatus*. Our results suggest that the 28 group II introns found in the *T. elongatus* genome arose from a single intron. An early event was deletion of the intron ORF, which appears to have occurred once prior to the divergence of intron families, giving rise to a smaller ORF-less intron that could be mobilized by the IEP of the intron from which it was derived. From that point, the ORF-containing and ORF-less introns diverged and proliferated in parallel by inserting into different sites, some of which were compatible with further mobility.

We identify four mechanisms that contributed to the proliferation of *T. elongatus* introns. First, we find that the *T. elongatus* introns have diverged into six families with different EBS sequences that target the introns to different sites. Although it had been thought that group II intron dispersal to new sites occurs primarily via retrotransposition, our results suggest instead that most of the *T. elongatus* introns have inserted into new sites via retrohoming after EBS sequence divergence. Sequence comparisons show that *T. elongatus* intron EBS sequences are relatively malleable compared to RNA regions required for ribozyme activity (Figure 2A). Mutations in the EBS sequences make it more difficult for an intron to splice and retrohome from its current site but increase its chances of retrohoming to a site not previously occupied by a related group II intron. Thus, this process enables waves of retrohoming into different sets of target sites, circumventing the problem of DNA target site saturation. As a proliferation mechanism, retrohoming has the selective advantage of ensuring that the intron inserts at sites with good EBS/IBS pairings from which it can subsequently splice efficiently, making intron insertion less deleterious to the host. By contrast, retrotransposition into essential genes with poorly matched exon sequences would be detrimental. Consequently, mutations leading to increased retrotransposition frequency may be lost by purifying selection, preventing this process from playing a greater role in group II intron proliferation.

A second proliferation mechanism is that some but not all *T. elongatus* IEPs have evolved to have relaxed intron specificity, enabling them to act as “driver” IEPs to mobilize multiple ORF-less introns with the same or greater efficiency than the intron in which they are encoded. Deletion of the intron ORF favors proliferation because the smaller, more compact ORF-less introns are less susceptible to nuclease degradation, which appears to be a major mechanism limiting the mobility of bacterial group II introns [35]. To mobilize an ORF-less intron by retrohoming or retrotransposition, a driver IEP must be able to splice the intron to generate RNPs that promote mobility. Thus, the dispersal of degenerate ORF-less introns by a driver IEP automatically leads to the evolution of a common splicing apparatus. Other bacteria have also been found to contain ORF-less introns that are spliced by the IEP of a closely related intron, but because the introns are very closely related, it has not been clear to what extent this ability involved relaxation of IEP specificity [40]. A distinctive feature of the *T. elongatus* introns is that we identify one IEP, TeI4h, which retains high specificity for its cognate intron, presumably reflecting the ancestral situation, and other closely related IEPs, such as TeI4c, which have diverged to mobilize other introns as or more efficiently than their cognate intron. As the specific TeI4h and relaxed TeI4c IEPs have 87% sequence identity with only a few divergent regions (Figure S1), further comparisons may facilitate the identification of IEP features that dictate intron specificity.

A third mechanism favoring intron proliferation in *T. elongatus* is the evolution of some introns to insert at a conserved site in another mobile element, either an insertion sequence or another mobile group II intron. The latter configuration, known as a twintron, could lead to intron proliferation either by retromobility of the composite intron or by separate splicing and mobility of the outer and inner introns. Supporting the latter mechanism, we find that the TeI4c IEP can independently splice and mobilize the outer TeI4c intron and the inner ORF-less TeI3c intron of the twintron (Table 2). Further, in all the *T. elongatus* twintrons, the inner F3 introns are identical (i.e., no sequence differences), while the outer F1 introns have diverged (Figure S5), as expected for recent independent insertions of an F3 intron into previously dispersed F1 introns. A previous example of group II intron proliferation via twintron formation was found in the archaebacteria *Methanosarcina acetivorans*, although in this case the twintron structure differs in involving nested arrays formed by repeated insertion of one group II intron into another [41]. A consequence of twintrons is the progressive expansion of non-essential genomic regions providing new target sites for intron insertion.

Finally, we find that proliferation of the *T. elongatus* IEPs is favored by higher temperatures that promote DNA strand separation, facilitating access to DNA target sites and enabling their recognition almost entirely by base pairing of the intron RNA. For other mobile group II introns, IEP recognition of the distal 5′-exon region upstream of IBS2 is required for reverse splicing into double-stranded but not single-stranded DNA, implying a major role in DNA strand separation, while IEP recognition of the 3′ exon is not required for DNA strand separation but is critical for second-strand cleavage by the En domain [31],[42]. The IEP encoded by EcI5, a closely related mesophilic intron, stringently recognizes four bases in the distal 5′-exon region upstream of EBS2 and one base in the 3′ exon downstream of EBS3 [28]. By contrast, the *T. elongatus* intron IEPs analyzed here recognize at most 1–2 bases in the distal 5′-exon region and none in the 3′ exon, with the stringency of IEP recognition decreasing at higher temperatures. We note that these relaxed DNA target site requirements with minimal IEP recognition found in mobility assays in *E. coli* at 48°C agree closely with the sequences of intron-insertion sites in the *T. elongatus* genome, implying that in both organisms the DNA target site is recognized in an unwound or more readily unwound state at higher temperature. As a result, the T. *elongatus* introns have access to a larger number of potential target sites compatible with intron RNA base pairing than do group II introns whose IEPs have higher target specificity. The lack of recognition of 3′-exon residues by the *T. elongatus* IEPs could reflect that distal 5′-exon and EBS/IBS interactions are sufficient for site-specific second-strand cleavage, that En cleavage is not stringently site specific, or that the introns dispense with En cleavage and use a nascent strand at a DNA replication fork to prime reverse transcription [1],[2]. As the other three proliferative mechanisms identified here are also available to mesophilic introns, retromobility at high temperature may be a key factor enabling the *T. elongatus* introns to proliferate to higher copy number than other group II introns.

In order to proliferate to high copy number, the *T. elongatus* introns must overcome selective pressure against retromobility. Such selective pressure is evidenced by the accumulation of mutations that impair retromobility of some *T. elongatus* introns (e.g., the YAGD and δ-δ′ pairing mutations in TeI4h, multiple mutations that inactivate TeI4d and TeI4f, and insertion of an IS element into the ORF of TeI4g). Selection for mutations that decrease mobility is common for bacterial and organellar group II introns and presumably reflects that actively mobile introns are deleterious to the host because they can insert into essential genes or make harmful double-strand chromosome breaks [1],[2],[37]. The proliferative mechanisms evolved by *T. elongatus* introns enable them to partially overcome purifying selection, striking what is for them a more favorable balance toward intron accumulation.

Importantly, we find that TeI4h and other *T. elongatus* introns are not only actively mobile but also thermophilic (Figure 4B and Table 1), suggesting that both the intron RNA and IEP have structural adaptations for thermostability. The *T. elongatus* intron ribozymes have a higher GC content (55.3%–56.2% for TeI4h, 4c, and 3c) than does EcI5 (51.2%), possibly contributing to their greater stability at higher temperature. The actively mobile thermostable introns can potentially be used for structural analysis, as well as for practical applications. The latter include use of thermostable group II intron RTs for RT-PCR and next-generation RNA sequencing and use of the thermophilic introns as gene targeting vectors for thermophiles, patterned after “targetrons” developed from mesophilic group II introns that can be reprogrammed to insert at desired sites by modifying the base pairing sequences in the intron RNA [32],[37].

Finally, we speculate that the mechanisms elucidated here could have contributed to the initial proliferation of mobile group II introns in the nuclear genomes of ancestral eukaryotes. Estimates of paleotemperatures based on isotopic measurements and phylogenetic reconstruction of ancient enzymes suggest that eukaryotes evolved at a time of higher than present-day temperatures (50–65°C) [43]. By promoting DNA melting, elevated temperatures would facilitate access of group II introns to DNA target sites and increase the number of target sites that could be recognized by base pairing of the intron RNA, with little or no additional constraints for IEP recognition. Indeed, it is possible that ancestral group II introns evolved to insert by reverse splicing into RNA or single-stranded DNA at higher temperature and became dependent upon the IEP for DNA strand separation only after temperatures cooled. Another important factor in the initial intron invasion of eukaryotic genomes may have been the evolution of driver IEPs that could splice and mobilize multiple group II introns, enabling most introns to lose their own ORFs. In addition to providing a common splicing apparatus, the evolution of IEPs to function efficiently in *trans* rather than *cis* as for other mobile group II introns [44] would have been essential to support group II intron proliferation after evolution of the nuclear membrane, which separates transcription from translation. Ultimately, the evolution of the spliceosome, a common RNA-based catalytic machinery consisting of snRNAs derived from group II intron RNA domains, would have enabled more extensive intron degeneration, leaving only minimal recognition motifs for the splice sites and branch-point nucleotide. Coupled with the existence of a common splicing apparatus, such degeneration would have accelerated intron proliferation by increasing the chances that functional introns could arise *de novo* as a result of mutations or recombination events that introduce the minimal recognition sequences [45],[46].

## Materials and Methods

### Recombinant Plasmids

*T. elongatus* introns and flanking exons were cloned via PCR of *T. elongatus* BP-1 DNA provided by Dr. T. Kaneko, Kazusa DNA Research Institute, Japan. PCRs were done using Taq DNA polymerase (New England Biolabs, Ipswich, MA). For ORF containing introns, 5′ and 3′ segments of the intron were amplified separately by PCRs using an exon primer that appends a PstI, BamHI, or EcoRI site together with an intron primer that overlaps a unique site (HindIII or EcoRI). The PCR products were then cloned between compatible sites in the polylinker of pUC18 or 19.

Intron-donor plasmids for mobility assays were constructed in two steps to insert the coding sequences for the intron RNA followed by the IEP. In the first step, the *T. elongatus* introns were swapped for the Ll.LtrB intron/IEP cassette in donor plasmid pACD2X [47]. For ORF-containing *T. elongatus* introns, this was done via two PCRs that separately amplify 5′ and 3′ segments of the intron, while introducing a 1.5-kb deletion in the ORF coding sequence matching that in the ORF-less *T. elongatus* introns. These PCRs used end primers that append short flanking exons (∼15 nts) and unique cloning sites (SpeI (5′) and PstI and/or XhoI (3′)) and internal primers that replace the intron ORF in DIVb with a T7 promoter sequence and an MluI site. The two PCR products were then cloned between XbaI and XhoI sites of pADC2X. Twintrons were resolved by precisely deleting the internal intron using PCR primers spanning the insertion site and replacing the original fragment with the deleted one. The ORF-less *T. elongatus* introns were cloned directly into donor plasmid pACD2X by PCR of *T. elongatus* BP-1 DNA with primers that append SpeI and PstI sites. The T7 promoter and an MluI site were inserted into DIVb of the ORF-less introns by another round of PCR using the same external primer together with an internal primer that adds the T7 promoter and an MluI site and then swapping the PCR product back into pACD2X. Cloned introns were confirmed to have the sequence published for *T. elongatus* genomic DNA [26], except for TeI4c for which all clones differed from the published sequence by a single base (G1901A). In the second step of donor plasmid construction, the RT ORFs were amplified in two pieces from the pUC18 or 19 clones (see above) by PCR using a 5′ primer that appends a PstI site, phage T7 φ10 gene Shine-Dalgarno sequence, and ATG codon, a 3′ primer that appends an XhoI site, and internal primers that overlap a unique restriction site. The ORF was then cloned via a three-way ligation into the donor plasmid using the PstI and XhoI sites downstream of the previously inserted *T. elongatus* introns.

Donor plasmids with point mutations in TeI4h (T1G, C236T) and the TeI4h IEP (YAGD to YADD) were derived by PCR amplifying the wild-type intron or IEP of previously constructed plasmids with primers that introduce the modification and then swapping the PCR product containing the mutation for the wild-type sequence to generate donor plasmids containing different combinations of mutations. The TeI4f intron's EBS1, EBS2, and EBS3 sequences were modified by quick-change site-directed mutagenesis (Stratagene, La Jolla, CA) to GTTCTG, TTCAA, and A, respectively, in order to improve complementarity to IBS1, 2, and 3 in the 5′ and 3′ exons.

Recipient plasmids for the *T. elongatus* introns contain ligated-exon sequences (TeI4h, −46/+22; TeI4f, −50/+15; TeI4c, −40/+20; TeI3f, 3k, 3l, 3c, 4a, 4b, 4d, 4e, −30/+15) cloned between the PstI site (or AatII site for the TeI4f target) and the EcoRI site of pBRR-tet [36]. The intron target sites were made either by PCR of cloned exon sequences with primers that append PstI and EcoRI sites or by inserting two complementary oligonucleotides with flanking PstI and EcoRI sites.

Recipient plasmid libraries for TeI3c and 4c were constructed by starting with synthetic DNA oligonucleotides that contain randomized target site positions −30 to −12 or −13 and +2 to +20 with a 5′ PstI site and a 3′ EcoRI site followed by the 3′ sequence tag 5′ GAATTCGACAACCCAACAG. The opposite strand was then synthesized with Klenow DNA polymerase (New England Biolabs) using a primer complementary to the tag, and the resulting double-stranded DNA target site was cloned between the PstI and EcoRI sites of pBRR-tet. The recipient plasmid library for TeI4h was constructed similarly by using Klenow DNA polymerase to fill in annealed, overlapping oligonucleotides that contain the randomized 5′- or 3′-exon sequences and append PstI and EcoRI sites.

All constructs were confirmed by sequencing the PCR amplified or modified region.

### Intron Mobility Assays

Mobility assays were done in *E. coli* HMS174(DE3) (Novagen, Madison, WI) grown in LB medium, with antibiotics added as required at the following concentrations: ampicillin, 100 µg/ml; chloramphenicol, 25 µg/ml; tetracycline, 25 µg/ml. Cells, which had been co-transformed with the Cap^R^ donor and Amp^R^ recipient plasmids, were inoculated into 5 ml of LB medium containing chloramphenicol and ampicillin and grown with shaking (200 rpm) overnight at 37°C. A small portion (50 µl) of the overnight culture was inoculated into 5 ml of fresh LB medium containing the same antibiotics and grown for 1 h as above. The cells were then induced by adding 1 ml of fresh LB medium containing the same antibiotics and 3 mM IPTG (500 µM final) and incubating for 1 h at temperatures specified for individual experiments. In mobility assays with TeI4h and its mutant derivatives at 37°C, changing the IPTG concentration from 100 to 1,000 µM or induction time from 30 to 90 min gave at most a 2-fold increase in mobility efficiency. For determination of temperature dependence, the initial log-phase cultures (5 ml) grown at 37°C were mixed with an equal volume of fresh LB medium containing antibiotics and 1 mM IPTG (500 µM final) that had been pre-warmed to achieve the desired temperature. The cultures were then induced for 1 h at that temperature, placed on ice, diluted with ice-cold LB, and plated at different dilutions onto LB agar containing ampicillin or ampicillin plus tetracycline. After incubating the plates overnight at 37°C, the mobility efficiency was calculated as the ratio of (Tet^R^+Amp^R^)/Amp^R^ colonies.

To verify correct insertion at the target site, Tet^R^+Amp^R^ colonies were picked into duplicate 96-well plates and used for colony PCR to separately amplify the 5′- and 3′-integration junctions using the primers Rsense (5′-ACAAATAGGGGTTCCGCGCAC) plus Te680rc (5′-GTTGGTGACCGCACCAGT) for the 5′ junction and Te420f (5′-AACGCGGTAAGCCCGTA) plus Rev2pBRR (5′-AATGGACGATATCCCGCA) for the 3′ junction. The PCR products were purified with Sera-Mag magnetic beads (Seradyne, Indianapolis, IN) and sequenced using the primers TargetSeq (5′-ATGCGAGAGTAGGGAACTGC) for the 5′ junction and Te500f (5′-AAACCGTAAGGAATGCTGATG) or Te420f (see above) for the 3′ junction.

### Target-Site Determination

*E. coli* HMS174(DE3) that had been co-transformed with the Cap^R^ donor plasmid and an Amp^R^ recipient plasmid in which regions of the DNA target site had been randomized was induced with 500 µM IPTG for 1 h at the specified temperature and then plated on LB agar containing tetracycline and ampicillin, as described above for mobility assays. Tet^R^+Amp^R^ colonies were picked into 96-well plates for colony PCR and sequenced for target-site determination, as described above.

## Supporting Information

Figure S1 **Multiple amino acid sequence alignments of** ***T. elongatus*** **and other group II intron-encoded proteins.** The figure shows aligned predicted amino acid sequences of the IEPs of the group II introns TeI4a, b, c, e, f, g, and h, EcI5, and Ll.LtrB (LtrA protein). The boundaries of conserved RT sequence blocks and the X/thumb, DNA-binding, and DNA endonuclease domains are delineated above the aligned sequences. Identical amino acid residues in the *T. elongatus* group II IEPs are shown as white letters on a black background, and similar amino acid residues, based on the matrix of Henikoff and Henikoff [48], are highlighted by gray background. Dashes indicate gaps inserted to maximize sequence homology. The alignment was done with ClustalX [49] and refined manually.
(1.37 MB TIF)

Figure S2 **Alignment of DIV sequences of** ***T. elongatus*** **group II introns showing deletion breakpoints in the ORF-less introns.** The sequences are grouped by intron family and extend from the first to last nucleotide residue of the DIV stem. The positions of the AUG start and UGA stop codons of the intron ORF are indicated. The arrow at the top and asterisks in the alignment indicate the insertion site of TeI3c and other F3 introns in the TeI4a, b, c, d, e, and TeI3h introns. Bases identical in all sequences are in red. Regions with deletions or insertions in the ORF-less introns are highlighted in gray. All the ORF-less introns contain an additional U residue just downstream of the second inframe AUG of the RT ORF (highlighted in red). The alignments were done with ClustalX [49] and refined manually.
(2.38 MB TIF)

Figure S3 **Phylogeny of** ***T. elongatus*** **introns.** (A) Phylogram of full-length ORF-containing introns. (B) Phylogram of intron-encoded proteins. RNA and protein sequences were aligned with ClustalX [49], and the alignments were refined manually and used as input for Phylip (ver. 3.69, with default parameters [50]). The phylogenies were generated with program modules DNAdist and DNAcomp for nucleotide alignments or Protdist and Protcomp for IEP alignments using all of the Distance settings (F84, Kimura, Jukes-Cantor, LogDet) independently and varying the out-group (EcI5 or random Te intron). Trees were visualized with Treeview [51],[52] and were essentially the same regardless of distance or out-group settings. Support for the major groupings of the phylogram was obtained by bootstrapping 1,000 data sets (using Seqboot from Phylip ver. 3.69) and using these as input for DNAdist or Protdist. The output of the latter programs was then used to obtain consensus trees with Consense. The numbers indicate the percentage of times a particular grouping occurred in the 1,000 data sets.
(0.19 MB TIF)

Figure S4 Effect of mutation at DNA target site position C–16 on the mobility efficiency of TeI4h* at 37°C and 48°C. Mobility assays with donor plasmid TeI4h*/4h* and recipient plasmids containing either the wild-type or C–16G mutant DNA target site were done in *E. coli* at 37°C and 48°C, as described in Figure 4 and Materials and Methods. Donor plasmid expression was induced with 500 µM IPTG for 1 h, and mobility efficiencies were calculated as the ratio of (Tet^R^+Amp^R^)/Amp^R^ colonies. The bar graphs show the mobility efficiency with the C–16G mutant DNA target site normalized to that with the wild-type DNA target at 37°C and 48°C. Mobility efficiencies with the wild-type target site in these experiments were 11.5±1.7 and 98±3% at 37°C and 48°C, respectively. The data are the mean for three experiments, with the error bars indicating the standard deviation.
(0.02 MB TIF)

Figure S5 **Sequence alignment of F1 and F3 introns.** (A) Alignment of F1 ORF-containing introns. Mutations relative to the TeI4c sequence are indicated in red. (B) Alignment of F3 ORF-less introns. The alignments were done with ClustalX [49]. Identical nucleotides are indicated by an asterisk below the alignment.
(0.06 MB PDF)

Table S1 **Mobility efficiencies for donor plasmid TeI4c/4c with recipient plasmids containing different target sites.** Mobility assays were done in *E. coli*, as described in Figure 4 and Materials and Methods, using donor plasmid TeI4c/4c, which expresses the TeI4c-ΔORF intron and TeI4c IEP (2nd ATG), and recipient plasmids that contain target sites for the TeI4a, b, c, d, and e introns (i.e., ligated E1-E2 sequences flanking the intron-insertions sites in *T. elongatus*). Donor plasmid expression was induced with 500 µM IPTG for 1 h at 48°C. Mobility efficiencies were calculated as the ratio of (Tet^R^+Amp^R^)/Amp^R^ colonies and are the mean of two independent experiments. The two repeats for the TeI4b target site gave mobility efficiencies of 83% and 72%, and the variation between repeats for the other target sites was less than 3-fold. Similar results were obtained with the Te4Ic IEP expressed from the first ATG (not shown). Insertion of the intron at the expected target site was confirmed by PCR (12 colonies) and sequencing (3 colonies) in all cases, except for the TeI4d and TeI4e target sites, where 100% and 60%, respectively, of the colonies did not give the expected PCR product and thus may not correspond to be bona fide mobility events. In a related experiment, donor plasmid TeI3c/4c, which expresses the TeI3c intron and TeI4c IEP, likewise gave high mobility efficiencies (60%–80%) with alternative target sites (unpublished data).
(0.07 MB PDF)

## Abbreviations

Amp^R^: ampicillin resistant
Cap^R^: chloramphenicol resistant
CL: chloroplast-like
cp: chloroplast
E1: 5′ exon
E2: 3′ exon
EBS: exon-binding site
IBS: intron-binding site
IEP: intron-encoded protein
IPTG: isopropyl β-D-1-thiogalactopyranoside
LB medium: Luria-Bertani medium
ML: mitochondrial-like
mt: mitochondrial
ORF: open reading frame
RNP: ribonucleoprotein particle
RT: reverse transcriptase
Tet^R^: tetracycline resistant
