# Supplementary figures and images for: Mechanisms Used for Genomic Proliferation by Thermophilic Group II Introns
Source: PLoS Biol. 2010 Jun 8;8(6):e1000391. doi: 10.1371/journal.pbio.1000391 (PMC2882425; doi:10.1371/journal.pbio.1000391)

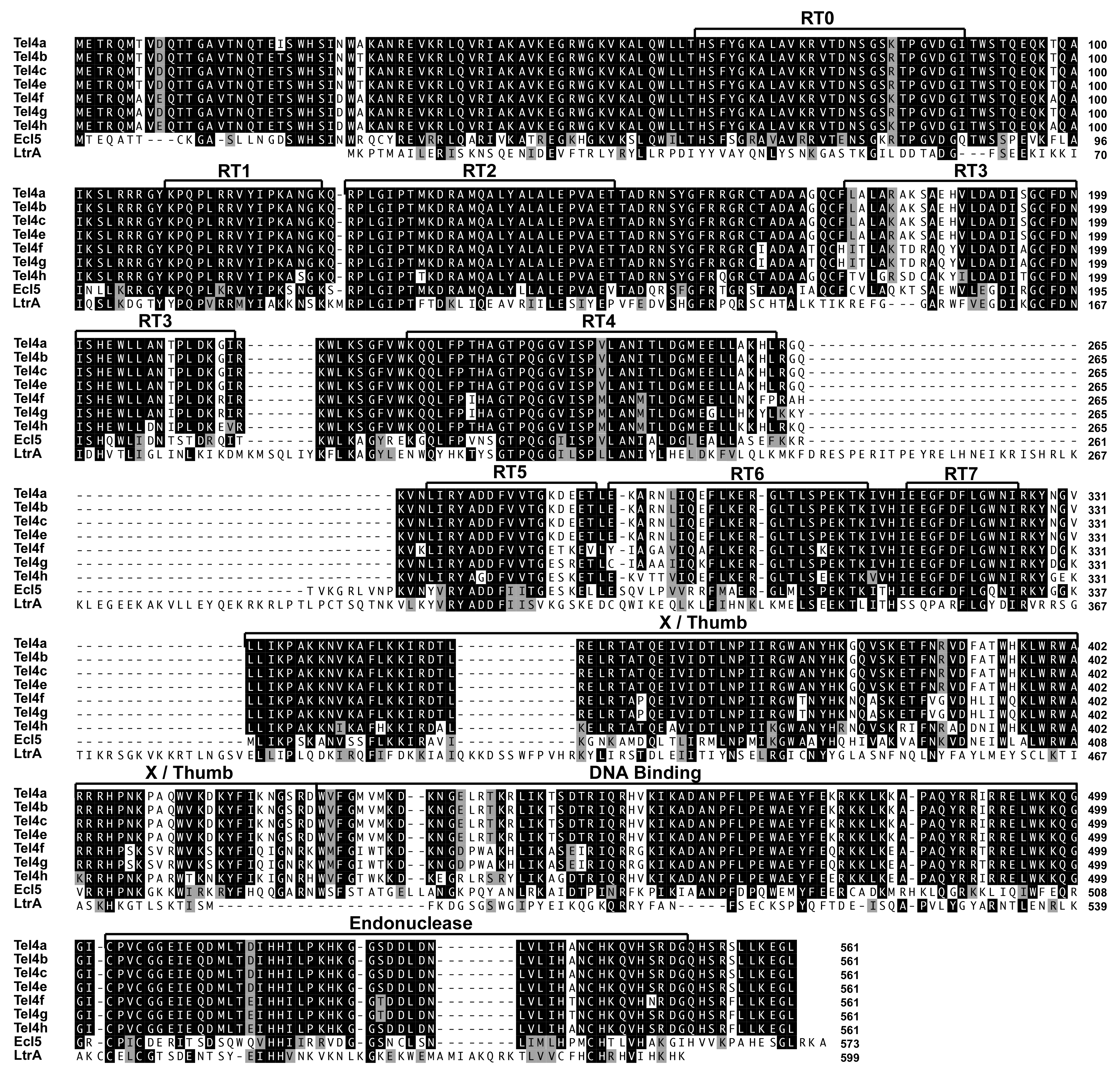

Supplement: Figure S1 — Multiple amino acid sequence alignments of T. elongatus and other group II intron-encoded proteins. The figure shows aligned predicted amino acid sequences of the IEPs of the group II introns TeI4a, b, c, e, f, g, and h, EcI5, and Ll.LtrB (LtrA protein). The boundaries of conserved RT sequence blocks and the X/thumb, DNA-binding, and DNA endonuclease domains are delineated above the aligned sequences. Identical amino acid residues in the T. elongatus group II IEPs are shown as white letters on a black background, and similar amino acid residues, based on the matrix of Henikoff and Henikoff [48], are highlighted by gray background. Dashes indicate gaps inserted to maximize sequence homology. The alignment was done with ClustalX [49] and refined manually. (1.37 MB TIF) [file pbio.1000391.s001.tif]

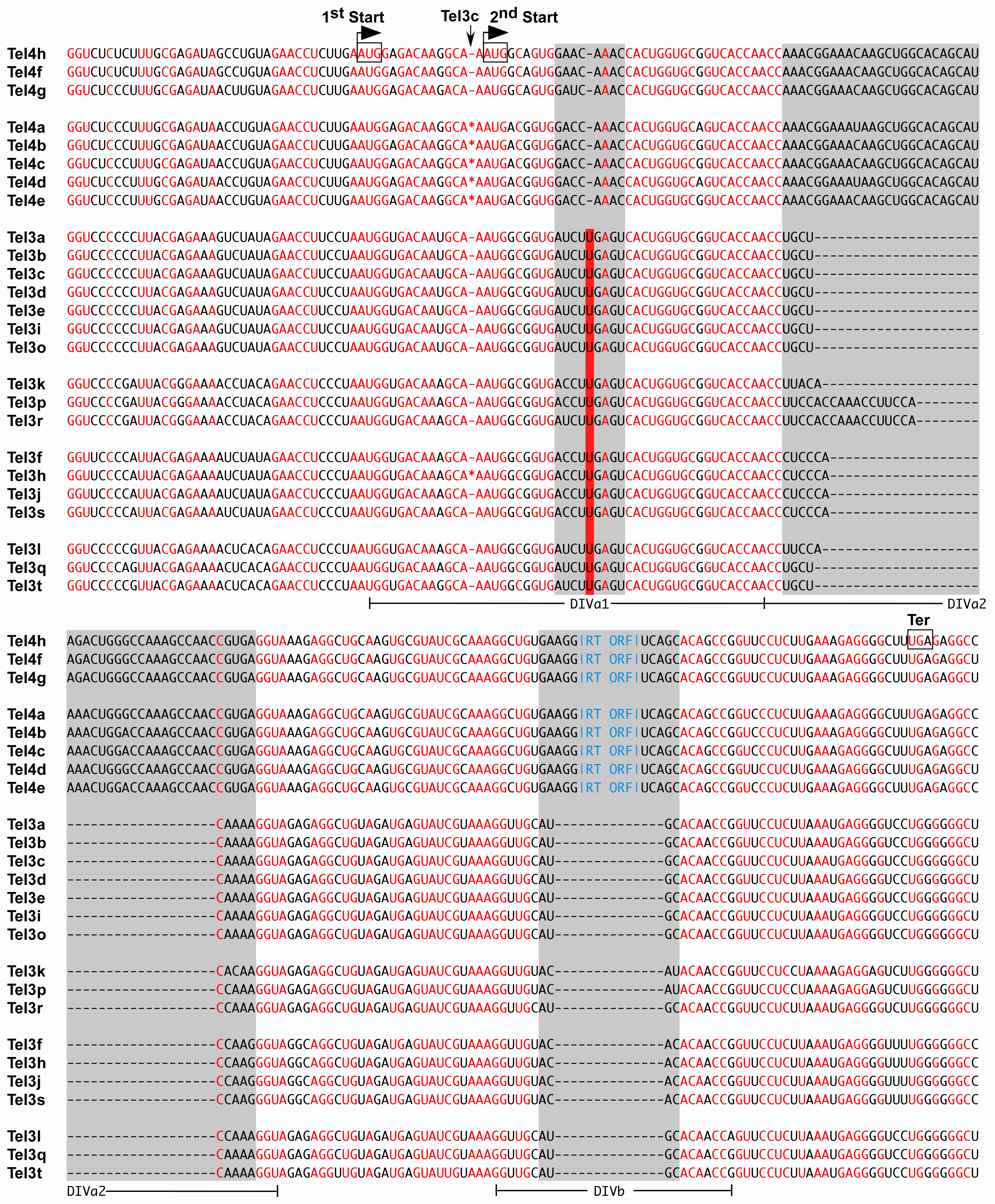

Supplement: Figure S2 — Alignment of DIV sequences of T. elongatus group II introns showing deletion breakpoints in the ORF-less introns. The sequences are grouped by intron family and extend from the first to last nucleotide residue of the DIV stem. The positions of the AUG start and UGA stop codons of the intron ORF are indicated. The arrow at the top and asterisks in the alignment indicate the insertion site of TeI3c and other F3 introns in the TeI4a, b, c, d, e, and TeI3h introns. Bases identical in all sequences are in red. Regions with deletions or insertions in the ORF-less introns are highlighted in gray. All the ORF-less introns contain an additional U residue just downstream of the second inframe AUG of the RT ORF (highlighted in red). The alignments were done with ClustalX [49] and refined manually. (2.38 MB TIF) [file pbio.1000391.s002.tif]

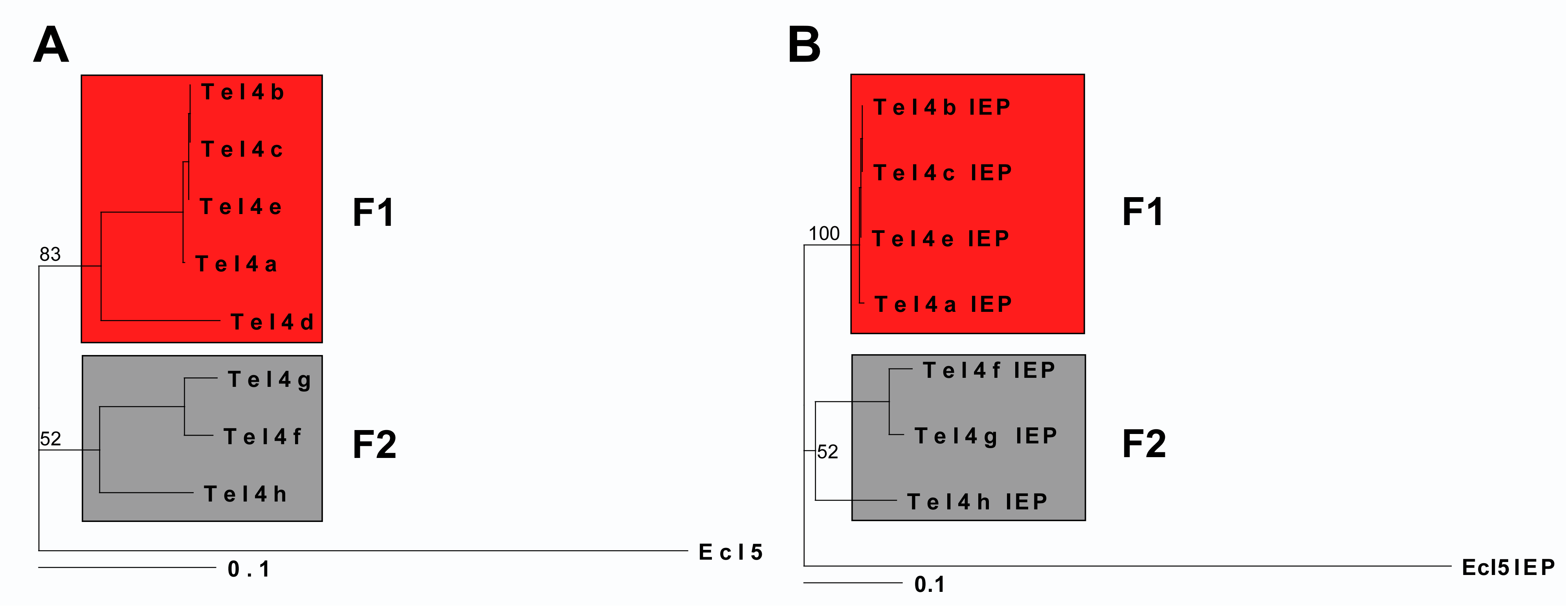

Supplement: Figure S3 — Phylogeny of T. elongatus introns. (A) Phylogram of full-length ORF-containing introns. (B) Phylogram of intron-encoded proteins. RNA and protein sequences were aligned with ClustalX [49], and the alignments were refined manually and used as input for Phylip (ver. 3.69, with default parameters [50]). The phylogenies were generated with program modules DNAdist and DNAcomp for nucleotide alignments or Protdist and Protcomp for IEP alignments using all of the Distance settings (F84, Kimura, Jukes-Cantor, LogDet) independently and varying the out-group (EcI5 or random Te intron). Trees were visualized with Treeview [51],[52] and were essentially the same regardless of distance or out-group settings. Support for the major groupings of the phylogram was obtained by bootstrapping 1,000 data sets (using Seqboot from Phylip ver. 3.69) and using these as input for DNAdist or Protdist. The output of the latter programs was then used to obtain consensus trees with Consense. The numbers indicate the percentage of times a particular grouping occurred in the 1,000 data sets. (0.19 MB TIF) [file pbio.1000391.s003.tif]
