# Supplementary material for: Mechanisms Used for Genomic Proliferation by Thermophilic Group II Introns
Source: PLoS Biol. 2010 Jun 8;8(6):e1000391. doi: 10.1371/journal.pbio.1000391 (PMC2882425; doi:10.1371/journal.pbio.1000391)
Supplement: Figure S5 — Sequence alignment of F1 and F3 introns. (A) Alignment of F1 ORF-containing introns. Mutations relative to the TeI4c sequence are indicated in red. (B) Alignment of F3 ORF-less introns. The alignments were done with ClustalX [49]. Identical nucleotides are indicated by an asterisk below the alignment. (0.06 MB PDF) [file pbio.1000391.s005.pdf]

Fig. S5

A. Family F1 alignment

```
Tel4d  GTGCGATGCGAAAGCCAGCCAGGTGATTGTCCCGTTAGCCCAACAAGCTAGAACGGGACCGATTGTTCCCCCAACCGTAGCCTAGGGAGGCATGCGCGAC  100
Tel4a  GTGCGACGCGAAAGCTAGCCAGATGATTGTCCCACTAGCCCAACAAGCTAGAACGGGACCGGTTGTTCCCCCAACCGTAGCCTAGGGAGGCATGCGCGAC  100
Tel4b  GTGCGACGCGAAAGCTAGCCAGATGATTGTCCCACTAGCCCAACAAGCTAGAACGGGACCGGTTGTTCCCCCAACCGTAGCCTAGGGAGGCATGCGCGAC  100
Tel4c  GTGCGACGCGAAAGCTAGCCAGATGATTGTCCCACTAGCCCAACAAGCTAGAACGGGACCGGTTGTTCCCCCAACCGTAGCCTAGGGAGGCATGCGCGAC  100
Tel4e  GTGCGACGCGAAAGCTAGCCAGATGATTGTCCCACTAGCCCAACAAGCTAGAACGGGACCGGTTGTTCCCCCAACCGTAGCCTAGGGAGGCATGCGCGAC  100
       ****** ******** ****** **********  ************************** **************************************

Tel4d  TGGTAACGGTCAGGTATGAAGCCCTCCCGACAACGGAGCCCGAACCGAAAGGTTGAAGCCGAATCCGTGAGGAGGAAGCAACTTTGCCAGCGTCAGGCGA  200
Tel4a  TGGTAACGGTCAGGTATGAAGCCCTCCCGACAACGGAGCCCGAACCGAAAGGTTGAAGCCGAATCCGTGAGGAGGAAGCAACTTTGCCAGCGTCAGGCGA  200
Tel4b  TGGTAACGGTCAGGTATGAAGCCCTCCCGACAACGGAGCCCGAACCGAAAGGTTGAAGCCGAATCCGTGAGGAGGAAGCAACTTTGCCAGCGTCAGGCGA  200
Tel4c  TGGTAACGGTCAGGTATGAAGCCCTCCCGACAACGGAGCCCGAACCGAAAGGTTGAAGCCGAATCCGTGAGGAGGAAGCAACTTTGCCAGCGTCAGGCGA  200
Tel4e  TGGTAACGGTCAGGTATGAAGCCCTCCCGACAACGGAGCCCGAACCGAAAGGTTGAAGCCGAATCCGTGAGGAGGAAGCAACTTTGCCAGCGTCAGGCGA  200
       ****************************************************************************************************

Tel4d  TAGGGAGCTAGGCTTGAGGGTATGGTGAACGCAAGTGAAGTGACGCCAGAAGCCTCGTTACTATAAACAGGCCAAAGACGCCGATAGGCCTGAGCCAAAA  300
Tel4a  TAGGGAGCTAGGCTTGAGGGTATGGTGAACGCAAGTGAAGTGACGCCAGAAGCCTCGTTACTATAAACAGGCCAAAGACGCCGATAGGCCTGAGCCAAAA  300
Tel4b  TAGGGAGCTAGGCTTGAGGGTATGGTGAACGCAAGTGAAGTGACGCCAGAAGCCTCGTTACTATAAACAGGCCAAAGACGCCGATAGGCCTGAGCCAAAA  300
Tel4c  TAGGGAGCTAGGCTTGAGGGTATGGTGAACGCAAGTGAAGTGACGCCAGAAGCCTCGTTACTATAAACAGGCCAAAGACGCCGATAGGCCTGAGCCAAAA  300
Tel4e  TAGGGAGCTAGGCTTGAGGGTATGGTGAACGCAAGTGAAGTGACGCCAGAAGCCTCGTTACTATAAACAGGCCAAAGACGCCGATAGGCCTGAGCCAAAA  300
       ****************************************************************************************************

Tel4d  TGGCAAACGGACTGGTTTCACTGCTGTCATCTCAGTGGACGGGGACAACAGCTCCGCCGGGGTAAAGTCACCACCTAACCCCTCGCGTCATCTGGTTGGA  400
Tel4a  TGGCAAACGGACTGGTTTCACTGCTGTCATCTCAGTGGACGGGGACAATAGTTCCGTCGGGGTAAAGTCACCACCTAACCCCTCGCGTCATCTGGTTGGA  400
Tel4b  TGGCAAACGGACTGGTTTCACTGCTGTCATCTCAGTGGACGGGGACAATAGTTCCGTCGGGGTAAAGTCACCACCTAACCCCTCGCGTCATCTGGTTGGA  400
Tel4c  TGGCAAACGGACTGGTTTCACTGCTGTCATCTCAGTGGACGGGGACAATAGTTCCGTCGGGGTAAAGTCACCACCTAACCCCTCGCGTCATCTGGTTGGA  400
Tel4e  TGGCAAACGGACTGGTTTCACTGCTGTCATCTCAGTGGACGGGGACAATAGTTCCGTCGGGGTAAAGTCACCACCTAACCCCTCGCGTCATCTGGTTGGA  400
       ************************************************ ** **** *******************************************

Tel4d  ACGCGGTAAGCCCGTATCGTCGCCTTGAACATTCAAGGCAGGCAAACCGTAAGGAATGCTGATGAGGGTGCGGGTATGGGATGCAGGAGAAAGCGAATGC  500
Tel4a  ACGCGGTAAGCCCGTATCTTCGCCTTGAACATTCAAGGCAGGCAAACCGTAAGGAATGCTGATGAGGGTGCGGGTATGGGATGCAGGAGAAAGCGAATGC  500
Tel4b  ACGCGGTAAGCCCGTATCTTCGCCTTGAACATTCAAGGCAGGCAAACCGTAAGGAATGCTGATGAGGGTGCGGGTATGGGATGCAGGAGAAAGCGAATGC  500
Tel4c  ACGCGGTAAGCCCGTATCTTCGCCTTGAACATTCAAGGCAGGCAAACCGTAAGGAATGCTGATGAGGGTGCGGGTATGGGATGCAGGAGAAAGCGAATGC  500
Tel4e  ACGCGGTAAGCCCGTATCTTCGCCTTGAACATTCAAGGCAGGCAAACCGTAAGGAATGCTGATGAGGGTGCGGGTATGGGATGCAGGAGAAAGCGAATGC  500
       ****************** *********************************************************************************

Tel4d  TGGTCTGTAACGGACCGGATAGGGGTTGAGGAGAGGACCCAACATCACCCCGCCCGAAAGGGAGCAGACTTCCTGCTGGTCTCCCTTTGCGAGATAACCT  600
Tel4a  TGGTCTGTAACGGACCGGATAGGGGTTGAGGAAACAATCCAACATCACCCCGCCCGAAAGGGAGCAGACTTCCTGCTGGTCTCCCTTTGCGAGATAACCT  600
Tel4b  TGGTCTGTAACGGACCGGATAGGGGTTGAGGAAACAATCCAACATCACCCCGCCCGAAAGGGAGCAGACTTCCTGCTGGTCTCCCTTTGCGAGATAACCT  600
Tel4c  TGGTCTGTAACGGACCGGATAGGGGTTGAGGAAACAATCCAACATCACCCCGCCCGAAAGGGAGCAGACTTCCTGCTGGTCTCCCTTTGCGAGATAACCT  600
Tel4e  TGGTCTGTAACGGACCGGATAGGGGTTGAGGAAACAATCCAACATCACCCCGCCCGAAAGGGAGCAGACTTCCTGCTGGTCTCCCTTTGCGAGATAACCT  600
       ******************************** *  * **************************************************************

Tel4d  GTAGAACCTCTTGAATGGAGACAAGGCAAATGACGGTGGACCAAACCACTGGTGCAGTCACCAACCAAACGGAAATAAGCTGGCACAGCATAAACTGGGC  700
Tel4a  GTAGAACCTCTTGAATGGAGACAAGGCAAATGACGGTGGACCAAACCACTGGTGCAGTCACCAACCAAACGGAAATAAGCTGGCACAGCATAAACTGGGC  700
Tel4b  GTAGAACCTCTTGAATGGAGACAAGGCAAATGACGGTGGACCAAACCACTGGTGCGGTCACCAACCAAACGGAAACAAGCTGGCACAGCATAAACTGGAC  700
Tel4c  GTAGAACCTCTTGAATGGAGACAAGGCAAATGACGGTGGACCAAACCACTGGTGCGGTCACCAACCAAACGGAAACAAGCTGGCACAGCATAAACTGGAC  700
Tel4e  GTAGAACCTCTTGAATGGAGACAAGGCAAATGACGGTGGACCAAACCACTGGTGCGGTCACCAACCAAACGGAAACAAGCTGGCACAGCATAAACTGGAC  700
       ******************************************************* ******************* ********************** *

Tel4d  CAAAGCCAACCGTGAGGTAAAGAGGCTGCAAGTGCGTATCGCAAAGGCTGTGAAGGAAGGACGCTGGGGCAAAGTGAAAGCTTTGCAATGGCTCCTGACC  800
Tel4a  CAAAGCCAACCGTGAGGTAAAGAGGCTGCAAGTGCGTATCGCAAAGGCTGTGAAGGAAGGACGCTGGGGCAAAGTGAAAGCTTTGCAATGGCTCCTGACC  800
Tel4b  CAAAGCCAACCGTGAGGTAAAGAGGCTGCAAGTGCGTATCGCAAAGGCTGTGAAGGAAGGACGCTGGGGCAAAGTGAAAGCTTTGCAATGGCTCCTGACC  800
Tel4c  CAAAGCCAACCGTGAGGTAAAGAGGCTGCAAGTGCGTATCGCAAAGGCTGTGAAGGAAGGACGCTGGGGCAAAGTGAAAGCTTTGCAATGGCTCCTGACC  800
Tel4e  CAAAGCCAACCGTGAGGTAAAGAGGCTGCAAGTGCGTATCGCAAAGGCTGTGAAGGAAGGACGCTGGGGCAAAGTGAAAGCTTTGCAATGGCTCCTGACC  800
       ****************************************************************************************************

Tel4d  CACTCGTTCTACGGCAAAGCCCTTGCCGTGAAACGGGTAACTGACAACTCAGGCAGTAAAACACCTGGTGTGGATGGGATAACCTGGTCCACACAAGAGC  900
Tel4a  CACTCGTTCTACGGCAAAGCCCTTGCCGTGAAACGGGTAACTGACAACTCAGGCAGTAAAACACCTGGTGTGGATGGGATAACCTGGTCCACACAAGAGC  900
Tel4b  CACTCGTTCTACGGCAAAGCCCTCGCCGTGAAACGGGTAACTGACAACTCAGGCAGTAGAACACCTGGTGTGGACGGGATAACCTGGTCCACACAAGAGC  900
Tel4c  CACTCGTTCTACGGCAAAGCCCTCGCCGTGAAACGGGTAACTGACAACTCAGGCAGTAGAACACCTGGTGTGGACGGGATAACCTGGTCCACACAAGAGC  900
Tel4e  CACTCGTTCTACGGCAAAGCCCTCGCCGTGAAACGGGTAACTGACAACTCAGGCAGTAGAACACCTGGTGTGGACGGGATAACCTGGTCCACACAAGAGC  900
       *********************** ********************************** *************** *************************

Tel4d  AGAAAACCCAAGCCATAAAGTCCCTCAGGAGAAGAGGCTATAAGCCCCAACCCCTGAGGCGGGTATATATCCCGAAAGCAAACGGCAAACAGCGCCCGCT  1000
Tel4a  AGAAAACCCAAGCCATAAAGTCCCTCAGGAGAAGAGGCTATAAGCCCCAACCCCTGAGGCGGGTATATATCCCGAAAGCAAACGGCAAACAGCGCCCGCT  1000
Tel4b  AGAAAACCCAAGCCATAAAGTCCCTCAGGAGAAGAGGCTATAAACCCCAACCCCTGAGGCGGGTATACATCCCGAAAGCAAACGGCAAACAGCGCCCGCT  1000
Tel4c  AGAAAACCCAAGCCATAAAGTCCCTCAGGAGAAGAGGCTATAAACCCCAACCCCTGAGGCGGGTATACATCCCGAAAGCAAACGGCAAACAGCGCCCGCT  1000
Tel4e  AGAAAACCCAAGCCATAAAGTCCCTCAGGAGAAGAGGCTATAAACCCCAACCCCTGAGGCGGGTATACATCCCGAAAGCAAACGGCAAACAGCGCCCGCT  1000
       ******************************************* *********************** ********************************

Tel4d  AGGAATCCCGACAATGAGGGACAGGGCAATGCAGGCACTATATGCACTAGCCCTAGAACCAGTCGCGGAAACCACAGCGGACCGGAACTCCTATGGGTTC  1100
Tel4a  AGGAATCCCGACAATGAAGGACAGGGCAATGCAGGCACTATATGCACTAGCCCTAGAACCAGTCGCGGAAACCACAGCAGACCGGAACTCCTATGGGTTC  1100
Tel4b  AGGAATCCCGACAATGAAGGACAGGGCAATGCAGGCACTATATGCCCTAGCCCTAGAACCAGTCGCGGAAACCACAGCGGACCGGAACTCCTATGGGTTC  1100
Tel4c  AGGAATCCCGACAATGAAGGACAGGGCAATGCAGGCACTATATGCCCTAGCCCTAGAACCAGTCGCGGAAACCACAGCGGACCGGAACTCCTATGGGTTC  1100
Tel4e  AGGAATCCCGACAATGAAGGACAGGGCAATGCAGGCACTATATGCACTAGCCCTAGAACCAGTCGCGGAAACCACAGCAGACCGGAACTCCTATGGGTTC  1100
       ***************** *************************** ********************************* ********************

Tel4d  CGGCGAGGACGTAGCACTGCAGACGCCGCCGGACAATGCTTTATAACACTAGCAAGGGCCGACAGCGCAACCTATGTCCCCGACGCTGATATCTCCGGAT  1200
Tel4a  CGCCGAGGGCGATGTACGGCAGATGCGGCAGGACAATGCTTCCTTGCTCTGGCAAGAGCCAAGTCGGCTGAACACGTCCTTGACGCTGACATATCCGGAT  1200
Tel4b  CGCCGAGGGCGATGTACGGCAGATGCGGCAGGACAATGCTTCCTTGCTCTGGCAAAAGCCAAGTCGGCTGAACACGTCCTTGACGCTGACATATCCGGAT  1200
Tel4c  CGCCGAGGGCGATGTACGGCAGATGCGGCAGGACAATGCTTCCTTGCTCTGGCAAAAGCCAAGTCGGCTGAACACGTCCTTGACGCTGACATATCCGGAT  1200
Tel4e  CGCCGAGGGCGATGTACGGCAGATGCGGCAGGACAATGCTTCCTTGCTCTGGCAAGAGCCAAGTCGGCTGAACACGTCCTTGACGCTGACATATCCGGAT  1200
       ** ***** **  * ** ***** ** ** ***********  *  * ** ****  *** *    **     * ****  ******** ** *******
```

```
Tel4d  GCTTTGACAATATCAGCCATGAGTGGCTACTAGCAAACATTCCACTTGACAAGGAAATCCTACGGAAGTGGCTCAAATCCGGGTTTGTCTGGAAACAGCA 1300
Tel4a  GCTTTGATAACATCAGCCATGAGTGGCTACTAGCCAACACTCCACTGGACAAAGGGATCTTACGGAAATGGCTTAAATCTGGGTTCGTCTGGAAACAGCA 1300
Tel4b  GCTTTGATAACATCAGCCATGAGTGGCTACTAGCCAACACTCCACTGGACAAAGGGATCTTACGGAAATGGCTTAAATCTGGGTTCGTCTGGAAACAGCA 1300
Tel4c  GCTTTGATAACATCAGCCATGAGTGGCTACTAGCCAACACTCCACTGGACAAAGGGATCTTACGGAAATGGCTTAAATCTGGGTTCGTCTGGAAACAGCA 1300
Tel4e  GCTTTGATAACATCAGCCATGAGTGGCTACTAGCCAACACTCCACTGGACAAAGGGATCTTACGGAAATGGCTTAAATCTGGGTTCGTCTGGAAACAGCA 1300
       ******* ** *********************** **** ****** ***** *   *** ******* ***** ***** ***** **************

Tel4d  ACTCTTCCCCACCCATGCTGGGACACCCCAGGGAGGGGTAATCTCCCCGATATTAGCAAACATGGCTCTGGATGGGATGGAAGAGCTACTGAATGAAGCA 1400
Tel4a  ACTCTTCCCCACCCATGCTGGGACACCTCAGGGAGGGGTAATCTCCCCAGTTCTTGCCAATATAACCCTAGATGGGATGGAAGAACTGTTGGCC-AAACA 1399
Tel4b  ACTCTTCCCCACCCATGCTGGGACACCTCAGGGAGGGGTAATCTCCCCAGTTCTTGCCAATATAACCCTAGATGGGATGGAAGAACTGTTGGCC-AAACA 1399
Tel4c  ACTCTTCCCCACCCATGCTGGGACACCTCAGGGAGGGGTAATCTCCCCAGTTCTTGCCAATATAACCCTAGATGGGATGGAAGAACTGTTGGCC-AAACA 1399
Tel4e  ACTCTTCCCCACCCATGCTGGGACACCTCAGGGAGGGGTAATCTCCCCAGTTCTTGCCAATATAACCCTAGATGGGATGGAAGAACTGTTGGCC-AAACA 1399
       *************************** ********************   *   * ** ** **   * ** ************* **   **     ** **

Tel4d  CTTCAGAAAAGCCAAAGTCCATCTCATTAGATATGCAGACGACTTTGTCGTAACCGGACAGACGAGGGAGACACTGTGTATTGCGGCAGCAATAATACAG 1500
Tel4a  CCTCAGAGGTCAAAAAGTCAACCTCATCCGATATGCTGACGATTTTGTCGTGACGGGAAAAGATGAGGAAACCCTGGAGAAAGCCAGAAACCTAATCCAG 1499
Tel4b  CCTCAGAGGTCAAAAAGTCAACCTCATCCGATATGCTGACGATTTTGTCGTGACGGGAAAAGATGAGGAAACCCTGGAGAAAGCCAGAAACCTAATCCAG 1499
Tel4c  CCTCAGAGGTCAAAAAGTCAACCTCATCCGATATGCTGACGATTTTGTCGTGACGGGAAAAGATGAGGAAACCCTGGAGAAAGCCAGAAACCTAATCCAG 1499
Tel4e  CCTCAGAGGTCAAAAAGTCAACCTCATCCGATATGCTGACGATTTTGTCGTGACGGGAAAAGATGAGGAAACCCTGGAGAAAGCCAGAAACCTAATCCAG 1499
       * *****       ****** * *****  ******* ***** ******** ** *** *       *** ** ***    *  **    *     **** ***

Tel4d  AAATTCCTCAAGAAAAGGGGCCTTACCCTATCACCGGAAAAGACAAAAATCGTCCATATCGAAGAAGGGTTCGACTTTCTCGGATGAAACATCCGCAAGT 1600
Tel4a  GAGTTCCTAAAAGAACGGGGCTTGACCCTGTCCCCCGAGAAGACAAAAATCGTCCATATTGAGGAAGGCTTCGACTTTCTCGGATGGAACATTCGCAAGT 1599
Tel4b  GAGTTCCTAAAAGAACGGGGCTTGACCCTGTCCCCCGAGAAGACAAAAATCGTCCATATTGAGGAAGGCTTCGACTTTCTCGGATGGAACATTCGCAAGT 1599
Tel4c  GAGTTCCTAAAAGAACGGGGCTTGACCCTGTCCCCCGAGAAGACAAAAATCGTCCATATTGAGGAAGGCTTCGACTTTCTCGGATGGAACATTCGCAAGT 1599
Tel4e  GAGTTCCTAAAAGAACGGGGCTTGACCCTGTCCCCCGAGAAGACAAAAATCGTCCATATTGAGGAAGGCTTCGACTTTCTCGGATGGAACATTCGCAAGT 1599
        * ***** **  ** ***** * ***** ** ** ** ******************* ** ***** **************** ***** *******

Tel4d  ACAAAGGAACACTCCTCATCAAACCTGCGGAAAAGAATATAAAAGCGTTCCCCAAGAAAATCCGAGACACACTGAAGCAACTTACAACAGCAACCCAAGA 1700
Tel4a  ACAACGGGGTTCTTCTCATCAAACCCGCGAAGAAGAACGTGAAAGCGTTCCTCAAGAAAATCCGAGACACTCTAAGGGAACTTAGGACAGCAACCCAGGA 1699
Tel4b  ACAACGGGGTTCTTCTCATCAAACCCGCGAAGAAGAACGTGAAAGCGTTCCTCAAGAAAATCCGAGACACTCTAAGGGAACTTAGGACAGCAACCCAGGA 1699
Tel4c  ACAACGGGGTTCTTCTCATCAAACCCGCGAAGAAGAACGTGAAAGCGTTCCTCAAGAAAATCCGAGACACTCTAAGGGAACTTAGGACAGCAACCCAGGA 1699
Tel4e  ACAACGGGGTTCTTCTCATCAAACCCGCGAAGAAGAACGTGAAAGCGTTCCTCAAGAAAATCCGAGACACTCTAAGGGAACTTAGGACAGCAACCCAGGA 1699
       **** **     ** *********** *** * *****  * ********** ***************** ** * * ******   *********** **

Tel4d  AACTGTGATAGAAGTGCTCAACCCAATTATCAGGGGATGGGCTAACTATCACAAAGGCCAAGCATCCAAGAAAACCTTTGCCCAAGTGGACCACCTCATC 1800
Tel4a  AATCGTGATAGACACACTCAACCCAATCATTAGAGGTTGGGCCAACTATCACAAAGGACAAGTCTCTAAGGAAACCTTCAACCGAGTGGACTTCGCCACC 1799
Tel4b  AATCGTGATAGACACACTCAACCCAATCATTAGAGGTTGGGCCAACTATCACAAAGGACAAGTCTCTAAGGAAACCTTCAACCGAGTGGACTTCGCCACC 1799
Tel4c  AATCGTGATAGACACACTCAACCCAATCATTAGAGGTTGGGCCAACTATCACAAAGGACAAGTCTCTAAGGAAACCTTCAACCGAGTGGACTTCGCCACC 1799
Tel4e  AATCGTGATAGACACACTCAACCCAATCATTAGAGGTTGGGCCAACTATCACAAAGGACAAGTCTCTAAGGAAACCTTCAACCGAGTGGACTTCGCCACC 1799
       **  ********     *********** ** ** ** ***** ************** ****  ** *** *******    ** *******  *  ** *

Tel4d  TGGCTAAAACTATGGCGATGGGCAAGGCGCCGGCACCCAAACAAACCACTCCGATGGGTGAAAAATAAATACATCATCCAAACCGGAAACAGAAACTGGG 1900
Tel4a  TGGCACAAATTGTGGCGATGGGCAAGGCGCCGGCACCCAAACAAACCTGCCCAATGGGTGAAGGACAAATACTTCATCAAAAACGGAAGCAGAGACTGGG 1899
Tel4b  TGGCACAAATTGTGGCGATGGGCAAGGCGCCGGCACCCAAACAAACCTGCCCAATGGGTGAAGGACAAATACTTCATCAAAAACGGAAGCAGAGACTGGG 1899
Tel4c  TGGCACAAATTGTGGCGATGGGCAAGGCGCCGGCACCCAAACAAACCTGCCCAATGGGTGAAGGACAAATACTTCATCAAAAACGGAAGCAGAGACTGGG 1899
Tel4e  TGGCACAAATTGTGGCGATGGGCAAGGCGCCGGCACCCAAACAAACCTGCCCAATGGGTGAAGGACAAATACTTCATCAAAAACGGAAGCAGAGACTGGG 1899
       ****  *** * ***********************************    ** *********  * ****** ***** *** ***** **** ******

Tel4d  TGTTCGGTATGCGGACGAAAGACAAGGATGGGAAACCGTGGGCTAAATACCTAACACAAGCCTCTGACACCCGAATCGAGCGCCACGTCAAAATCAAGGC 2000
Tel4a  TGTTCGGTATGGTGATGAAAGACAAGAACGGGGAACTGAGGACCAAACGCCTAATCAAAACCTCTGACACCCGAATCCAACGCCACGTCAAAATCAAGGC 1999
Tel4b  TGTTCGGTATGGTGATGAAAGACAAGAACGGGGAACTGAGGACCAAACGCCTAATCAAAACCTCTGACACCCGAATCCAACGCCACGTCAAAATCAAGGC 1999
Tel4c  TGTTCGGTATGGTGATGAAAGACAAGAACGGGGAACTGAGGACCAAACGCCTAATCAAAACCTCTGACACCCGAATCCAACGCCACGTCAAAATCAAGGC 1999
Tel4e  TGTTCGGTATGGTGATGAAAGACAAGAACGGGGAACTGAGGACCAAACGCCTAATCAAAACCTCTGACACCCGAATCCAACGCCACGTCAAAATCAAGGC 1999
       ***********  ** ********** * *** *** * ** * ***   *****    ** **************** * ********************

Tel4d  AGACGCCAACCCGTCTCTCCCAGAATGGACGGAATACTTT-AGAAACGCAAGAAACTCAAAGAAACCCCCGCCCAATACCGGTACACCCGCCGGAAACTA 2099
Tel4a  AGACGCCAATCCGTTTCTCCCAGAGTGGGCAGAATACTTTGAGAAACGCAAGAAACTCAAAAAAGCCCCTGCTCAATATCGGCGCATCCGCCGAGAACTA 2099
Tel4b  AGACGCCAATCCGTTTCTCCCAGAGTGGGCAGAATACTTTGAGAAACGCAAGAAACTCAAAAAAGCCCCTGCTCAATATCGGCGCATCCGCCGAGAACTA 2099
Tel4c  AGACGCCAATCCGTTTCTCCCAGAGTGGGCAGAATACTTTGAGAAACGCAAGAAACTCAAAAAAGCCCCTGCTCAATATCGGCGCATCCGCCGAGAACTA 2099
Tel4e  AGACGCCAATCCGTTTCTCCCAGAGTGGGCAGAATACTTTGAGAAACGCAAGAAACTCAAAAAAGCCCCTGCTCAATATCGGCGCATCCGCCGAGAACTA 2099
       ********* **** ********* *** * ********* ******************* ** **** ** ***** ***  ** ******  *****

Tel4d  TGGGAAAGACAAAATGGCACCTGCCCAGTATGCGGGGGAGAGATCGAACAAGACGATCTTACCGAAATCCACCACATACTGCCCAAACACAAGGGCGGTT 2199
Tel4a  TGGAAGAAACAGGGTGGTATCTGTCCAGTATGCGGGGGTGAAATTGAGCAAGACATGCTCACTGACATCCACCACATATTGCCCAAACACAAGGGTGGTT 2199
Tel4b  TGGAAGAAACAGGGTGGTATCTGTCCAGTATGCGGGGGTGAAATTGAGCAAGACATGCTCACTGACATCCACCACATATTGCCCAAACACAAGGGTGGTT 2199
Tel4c  TGGAAGAAACAGGGTGGTATCTGTCCAGTATGCGGGGGTGAAATTGAGCAAGACATGCTCACTGACATCCACCACATATTGCCCAAACACAAGGGTGGTT 2199
Tel4e  TGGAAGAAACAGGGTGGTATCTGTCCAGTATGCGGGGGTGAAATTGAGCAAGACATGCTCACTGACATCCACCACATATTGCCCAAACACAAGGGTGGTT 2199
       *** * * ***    *** * *** ************** ** ** ******    ** ** ** ************ **************** ****

Tel4d  CTGATGACCTGGACAACCTTGTCCTAATCCACGCCAACTGCCACAAACAGGTGCACAGCCAAGACGGTCAGCACAGCCGGTTCCTCTTGAAAGAGGGGCT 2299
Tel4a  CTGACGACCTGGATAATCTTGTCTTAATCCACGCCAACTGCCACAAACAGGTGCACAGCCGAGATGGTCAGCACAGCCGGTCCCTCTTGAAAGAGGGGCT 2299
Tel4b  CTGACGACCTGGATAATCTTGTCTTAATCCACGCCAACTGCCACAAACAGGTGCACAGCCGAGATGGTCAGCACAGCCGGTCCCTCTTGAAAGAGGGGCT 2299
Tel4c  CTGACGACCTGGATAATCTTGTCTTAATCCACGCCAACTGCCACAAACAGGTGCACAGCCGAGATGGTCAGCACAGCCGGTCCCTCTTGAAAGAGGGGCT 2299
Tel4e  CTGACGACCTGGATAATCTTGTCTTAATCCACGCCAACTGCCACAAACAGGTGCACAGCCGAGATGGTCAGCACAGCCGGTCCCTCTTGAAAGAGGGGCT 2299
       **** ******** ** ****** *********************************** *** *************** ******************

Tel4d  TTGAGAGGCTTGAGCCGGATGCGGGGAAACTGGCACGTCCGGTTCTTAGGGGGCTAGGGGGCAGTAATGCCCCCCCTGCTACCCGAC 2386
Tel4a  TTGAGAGGCCTGAGCCGGATGCTGGGAAACTAGCACGTCCGGTTCTTAGGGGGCTAGGGGGCAGTAATGCCCCCC--GCTACCCGAC 2384
Tel4b  TTGAGAGGCCTGAGCCGGATGCTGGGAAACTAGCACGTCCGGTTCTTAGGGGGCTAGGGGGCAGTAATGCCCCCC--GCTACCCGAC 2384
Tel4c  TTGAGAGGCCTGAGCCGGATGCTGGGAAACTAGCACGTCCGGTTCTTAGGGGGCTAGGGGGCAGTAATGCCCCCC--GCTACCCGAC 2384
Tel4e  TTGAGAGGCCTGAGCCGGATGCTGGGAAACTAGCACGTCCGGTTCTTAGGGGGCTAGGGGGCAGTAATGCCCCCC--GCTACCCGAC 2384
       ********* ************ ******** ******************************************   **********
```

B. Family F3 alignment

```
Tel3a   GTGCGACGCGAAAGCTAGCCAGATGATTGTCCCACTAGCCCAACAAGCTAGAACGGGACCGGTTGTTCCCCCAACCGTAGCCTAAGGAGGCATGCGTGAC 100
Tel3b   GTGCGACGCGAAAGCTAGCCAGATGATTGTCCCACTAGCCCAACAAGCTAGAACGGGACCGGTTGTTCCCCCAACCGTAGCCTAAGGAGGCATGCGTGAC 100
Tel3c   GTGCGACGCGAAAGCTAGCCAGATGATTGTCCCACTAGCCCAACAAGCTAGAACGGGACCGGTTGTTCCCCCAACCGTAGCCTAAGGAGGCATGCGTGAC 100
Tel3d   GTGCGACGCGAAAGCTAGCCAGATGATTGTCCCACTAGCCCAACAAGCTAGAACGGGACCGGTTGTTCCCCCAACCGTAGCCTAAGGAGGCATGCGTGAC 100
Tel3e   GTGCGACGCGAAAGCTAGCCAGATGATTGTCCCACTAGCCCAACAAGCTAGAACGGGACCGGTTGTTCCCCCAACCGTAGCCTAAGGAGGCATGCGTGAC 100
Tel3i   GTGCGACGCGAAAGCTAGCCAGATGATTGTCCCACTAGCCCAACAAGCTAGAACGGGACCGGTTGTTCCCCCAACCGTAGCCTAAGGAGGCATGCGTGAC 100
Tel3o   GTGCGACGCGAAAGCTAGCCAGATGATTGTCCCACTAGCCCAACAAGCTAGAACGGGACCGGTTGTTCCCCCAACCGTAGCCTAAGGAGGCATGCGTGAC 100
        ****************************************************************************************************

Tel3a   TGGTAACGGTCAGGTATGAAGCCCTCCCGACAATGAAGCCCGAACCGGAAGGTTGGAGCCGAATCCGTGAGGAGGAAGCAACTTCACCAGCGTCAGGTGA 200
Tel3b   TGGTAACGGTCAGGTATGAAGCCCTCCCGACAATGAAGCCCGAACCGGAAGGTTGGAGCCGAATCCGTGAGGAGGAAGCAACTTCACCAGCGTCAGGTGA 200
Tel3c   TGGTAACGGTCAGGTATGAAGCCCTCCCGACAATGAAGCCCGAACCGGAAGGTTGGAGCCGAATCCGTGAGGAGGAAGCAACTTCACCAGCGTCAGGTGA 200
Tel3d   TGGTAACGGTCAGGTATGAAGCCCTCCCGACAATGAAGCCCGAACCGGAAGGTTGGAGCCGAATCCGTGAGGAGGAAGCAACTTCACCAGCGTCAGGTGA 200
Tel3e   TGGTAACGGTCAGGTATGAAGCCCTCCCGACAATGAAGCCCGAACCGGAAGGTTGGAGCCGAATCCGTGAGGAGGAAGCAACTTCACCAGCGTCAGGTGA 200
Tel3i   TGGTAACGGTCAGGTATGAAGCCCTCCCGACAATGAAGCCCGAACCGGAAGGTTGGAGCCGAATCCGTGAGGAGGAAGCAACTTCACCAGCGTCAGGTGA 200
Tel3o   TGGTAACGGTCAGGTATGAAGCCCTCCCGACAATGAAGCCCGAACCGGAAGGTTGGAGCCGAATCCGTGAGGAGGAAGCAACTTCACCAGCGTCAGGTGA 200
        ****************************************************************************************************

Tel3a   TAGGGAGCTAGGCTTGAGGGTATGGTGAACGCAAGTGAAGTGACGCTAGAAGCCTCGTTACTCCAAGCAGGCCAAAGATGCTGATAGGCCTGAGCCAAAA 300
Tel3b   TAGGGAGCTAGGCTTGAGGGTATGGTGAACGCAAGTGAAGTGACGCTAGAAGCCTCGTTACTCCAAGCAGGCCAAAGATGCTGATAGGCCTGAGCCAAAA 300
Tel3c   TAGGGAGCTAGGCTTGAGGGTATGGTGAACGCAAGTGAAGTGACGCTAGAAGCCTCGTTACTCCAAGCAGGCCAAAGATGCTGATAGGCCTGAGCCAAAA 300
Tel3d   TAGGGAGCTAGGCTTGAGGGTATGGTGAACGCAAGTGAAGTGACGCTAGAAGCCTCGTTACTCCAAGCAGGCCAAAGATGCTGATAGGCCTGAGCCAAAA 300
Tel3e   TAGGGAGCTAGGCTTGAGGGTATGGTGAACGCAAGTGAAGTGACGCTAGAAGCCTCGTTACTCCAAGCAGGCCAAAGATGCTGATAGGCCTGAGCCAAAA 300
Tel3i   TAGGGAGCTAGGCTTGAGGGTATGGTGAACGCAAGTGAAGTGACGCTAGAAGCCTCGTTACTCCAAGCAGGCCAAAGATGCTGATAGGCCTGAGCCAAAA 300
Tel3o   TAGGGAGCTAGGCTTGAGGGTATGGTGAACGCAAGTGAAGTGACGCTAGAAGCCTCGTTACTCCAAGCAGGCCAAAGATGCTGATAGGCCTGAGCCAAAA 300
        ****************************************************************************************************

Tel3a   TGGCAAAGCGGATTGGATTCACTGCTTGCTTTTCAGTGAACGGGGACAGCAACTCCGCCGGGGTATAGTCACCACCTAACCCCTCGTGTCATCTGGTTGG 400
Tel3b   TGGCAAAGCGGATTGGATTCACTGCTTGCTTTTCAGTGAACGGGGACAGCAACTCCGCCGGGGTATAGTCACCACCTAACCCCTCGTGTCATCTGGTTGG 400
Tel3c   TGGCAAAGCGGATTGGATTCACTGCTTGCTTTTCAGTGAACGGGGACAGCAACTCCGCCGGGGTATAGTCACCACCTAACCCCTCGTGTCATCTGGTTGG 400
Tel3d   TGGCAAAGCGGATTGGATTCACTGCTTGCTTTTCAGTGAACGGGGACAGCAACTCCGCCGGGGTATAGTCACCACCTAACCCCTCGTGTCATCTGGTTGG 400
Tel3e   TGGCAAAGCGGATTGGATTCACTGCTTGCTTTTCAGTGAACGGGGACAGCAACTCCGCCGGGGTATAGTCACCACCTAACCCCTCGTGTCATCTGGTTGG 400
Tel3i   TGGCAAAGCGGATTGGATTCACTGCTTGCTTTTCAGTGAACGGGGACAGCAACTCCGCCGGGGTATAGTCACCACCTAACCCCTCGTGTCATCTGGTTGG 400
Tel3o   TGGCAAAGCGGATTGGATTCACTGCTTGCTTTTCAGTGAACGGGGACAGCAACTCCGCCGGGGTATAGTCACCACCTAACCCCTCGTGTCATCTGGTTGG 400
        ****************************************************************************************************

Tel3a   AACGCGGTAAGCCCGTATCTTCGCCTTGAACATTCAAGGCAGGCAAACCGTAAGGAATGCTGATGGGGGTGCGGGTAGAGGAGGTGGGAAAAAGCGAATG 500
Tel3b   AACGCGGTAAGCCCGTATCTTCGCCTTGAACATTCAAGGCAGGCAAACCGTAAGGAATGCTGATGGGGGTGCGGGTAGAGGAGGTGGGAAAAAGCGAATG 500
Tel3c   AACGCGGTAAGCCCGTATCTTCGCCTTGAACATTCAAGGCAGGCAAACCGTAAGGAATGCTGATGGGGGTGCGGGTAGAGGAGGTGGGAAAAAGCGAATG 500
Tel3d   AACGCGGTAAGCCCGTATCTTCGCCTTGAACATTCAAGGCAGGCAAACCGTAAGGAATGCTGATGGGGGTGCGGGTAGAGGAGGTGGGAAAAAGCGAATG 500
Tel3e   AACGCGGTAAGCCCGTATCTTCGCCTTGAACATTCAAGGCAGGCAAACCGTAAGGAATGCTGATGGGGGTGCGGGTAGAGGAGGTGGGAAAAAGCGAATG 500
Tel3i   AACGCGGTAAGCCCGTATCTTCGCCTTGAACATTCAAGGCAGGCAAACCGTAAGGAATGCTGATGGGGGTGCGGGTAGAGGAGGTGGGAAAAAGCGAATG 500
Tel3o   AACGCGGTAAGCCCGTATCTTCGCCTTGAACATTCAAGGCAGGCAAACCGTAAGGAATGCTGATGGGGGTGCGGGTAGAGGAGGTGGGAAAAAGCGAATG 500
        ****************************************************************************************************

Tel3a   CTAGCCTGTAATGGGCTAGATAGGGATTGAGAATGCTGGCAGGACATTCAACATCATCCCACTCGAAAGAGGGCAGACTTCCCGCCGGTCCCCCCTTACG 600
Tel3b   CTAGCCTGTAATGGGCTAGATAGGGATTGAGAATGCTGGCAGGACATTCAACATCATCCCACTCGAAAGAGGGCAGACTTCCCGCCGGTCCCCCCTTACG 600
Tel3c   CTAGCCTGTAATGGGCTAGATAGGGATTGAGAATGCTGGCAGGACATTCAACATCATCCCACTCGAAAGAGGGCAGACTTCCCGCCGGTCCCCCCTTACG 600
Tel3d   CTAGCCTGTAATGGGCTAGATAGGGATTGAGAATGCTGGCAGGACATTCAACATCATCCCACTCGAAAGAGGGCAGACTTCCCGCCGGTCCCCCCTTACG 600
Tel3e   CTAGCCTGTAATGGGCTAGATAGGGATTGAGAATGCTGGCAGGACATTCAACATCATCCCACTCGAAAGAGGGCAGACTTCCCGCCGGTCCCCCCTTACG 600
Tel3i   CTAGCCTGTAATGGGCTAGATAGGGATTGAGAATGCTGGCAGGACATTCAACATCATCCCACTCGAAAGAGGGCAGACTTCCCGCCGGTCCCCCCTTACG 600
Tel3o   CTAGCCTGTAATGGGCTAGATAGGGATTGAGAATGCTGGCAGGACATTCAACATCATCCCACTCGAAAGAGGGCAGACTTCCCGCCGGTCCCCCCTTACG 600
        ****************************************************************************************************

Tel3a   AGAAAGTCTATAGAACCTTCCTAATGGTGACAATGCAAATGGCGGTGATCTTGAGTCACTGGTGCGGTCACCAACCTGCTCAAAAGGTAGAGAGGCTGTA 700
Tel3b   AGAAAGTCTATAGAACCTTCCTAATGGTGACAATGCAAATGGCGGTGATCTTGAGTCACTGGTGCGGTCACCAACCTGCTCAAAAGGTAGAGAGGCTGTA 700
Tel3c   AGAAAGTCTATAGAACCTTCCTAATGGTGACAATGCAAATGGCGGTGATCTTGAGTCACTGGTGCGGTCACCAACCTGCTCAAAAGGTAGAGAGGCTGTA 700
Tel3d   AGAAAGTCTATAGAACCTTCCTAATGGTGACAATGCAAATGGCGGTGATCTTGAGTCACTGGTGCGGTCACCAACCTGCTCAAAAGGTAGAGAGGCTGTA 700
Tel3e   AGAAAGTCTATAGAACCTTCCTAATGGTGACAATGCAAATGGCGGTGATCTTGAGTCACTGGTGCGGTCACCAACCTGCTCAAAAGGTAGAGAGGCTGTA 700
Tel3i   AGAAAGTCTATAGAACCTTCCTAATGGTGACAATGCAAATGGCGGTGATCTTGAGTCACTGGTGCGGTCACCAACCTGCTCAAAAGGTAGAGAGGCTGTA 700
Tel3o   AGAAAGTCTATAGAACCTTCCTAATGGTGACAATGCAAATGGCGGTGATCTTGAGTCACTGGTGCGGTCACCAACCTGCTCAAAAGGTAGAGAGGCTGTA 700
        ****************************************************************************************************

Tel3a   GATGAGTATCGTAAAGGTTGCATGCACAACCGGTTCCTCTTAAATGAGGGGTCCTGGGGGGCTCGAGCCGGATGCGGGGAAACTTGCACGTCCGGTTCCT 800
Tel3b   GATGAGTATCGTAAAGGTTGCATGCACAACCGGTTCCTCTTAAATGAGGGGTCCTGGGGGGCTCGAGCCGGATGCGGGGAAACTTGCACGTCCGGTTCCT 800
Tel3c   GATGAGTATCGTAAAGGTTGCATGCACAACCGGTTCCTCTTAAATGAGGGGTCCTGGGGGGCTCGAGCCGGATGCGGGGAAACTTGCACGTCCGGTTCCT 800
Tel3d   GATGAGTATCGTAAAGGTTGCATGCACAACCGGTTCCTCTTAAATGAGGGGTCCTGGGGGGCTCGAGCCGGATGCGGGGAAACTTGCACGTCCGGTTCCT 800
Tel3e   GATGAGTATCGTAAAGGTTGCATGCACAACCGGTTCCTCTTAAATGAGGGGTCCTGGGGGGCTCGAGCCGGATGCGGGGAAACTTGCACGTCCGGTTCCT 800
Tel3i   GATGAGTATCGTAAAGGTTGCATGCACAACCGGTTCCTCTTAAATGAGGGGTCCTGGGGGGCTCGAGCCGGATGCGGGGAAACTTGCACGTCCGGTTCCT 800
Tel3o   GATGAGTATCGTAAAGGTTGCATGCACAACCGGTTCCTCTTAAATGAGGGGTCCTGGGGGGCTCGAGCCGGATGCGGGGAAACTTGCACGTCCGGTTCCT 800
        ****************************************************************************************************

Tel3a   AGGGGGCTAGGGGGCAGCGATGCCCCCCTGCTACCCGAC 839
Tel3b   AGGGGGCTAGGGGGCAGCGATGCCCCCCTGCTACCCGAC 839
Tel3c   AGGGGGCTAGGGGGCAGCGATGCCCCCCTGCTACCCGAC 839
Tel3d   AGGGGGCTAGGGGGCAGCGATGCCCCCCTGCTACCCGAC 839
Tel3e   AGGGGGCTAGGGGGCAGCGATGCCCCCCTGCTACCCGAC 839
Tel3i   AGGGGGCTAGGGGGCAGCGATGCCCCCCTGCTACCCGAC 839
Tel3o   AGGGGGCTAGGGGGCAGCGATGCCCCCCTGCTACCCGAC 839
        ***************************************
```
