# Supplementary material for: Mechanisms Used for Genomic Proliferation by Thermophilic Group II Introns
Source: PLoS Biol. 2010 Jun 8;8(6):e1000391. doi: 10.1371/journal.pbio.1000391 (PMC2882425; doi:10.1371/journal.pbio.1000391)
Supplement: Table S1 — Mobility efficiencies for donor plasmid TeI4c/4c with recipient plasmids containing different target sites. Mobility assays were done in E. coli, as described in Figure 4 and Materials and Methods, using donor plasmid TeI4c/4c, which expresses the TeI4c-ΔORF intron and TeI4c IEP (2nd ATG), and recipient plasmids that contain target sites for the TeI4a, b, c, d, and e introns (i.e., ligated E1-E2 sequences flanking the intron-insertions sites in T. elongatus). Donor plasmid expression was induced with 500 µM IPTG for 1 h at 48°C. Mobility efficiencies were calculated as the ratio of (TetR+AmpR)/AmpR colonies and are the mean of two independent experiments. The two repeats for the TeI4b target site gave mobility efficiencies of 83% and 72%, and the variation between repeats for the other target sites was less than 3-fold. Similar results were obtained with the Te4Ic IEP expressed from the first ATG (not shown). Insertion of the intron at the expected target site was confirmed by PCR (12 colonies) and sequencing (3 colonies) in all cases, except for the TeI4d and TeI4e target sites, where 100% and 60%, respectively, of the colonies did not give the expected PCR product and thus may not correspond to be bona fide mobility events. In a related experiment, donor plasmid TeI3c/4c, which expresses the TeI3c intron and TeI4c IEP, likewise gave high mobility efficiencies (60%–80%) with alternative target sites (unpublished data). (0.07 MB PDF) [file pbio.1000391.s006.pdf]

Table S1: Mobility Efficiencies for Donor Plasmid TeI4c/4c with Recipient Plasmids Containing Different Target Sites.

**Target site**	**Mobility (%)**
4a	$6.3x10^{-3}$
4b	78
4c	$1.5x10^{-2}$
4d	$2.2x10^{-4}$
4e	$4.6x10^{-4}$

Mobility efficiencies for donor plasmid TeI4c/4c with recipient plasmids containing different target sites. Mobility assays were done in *E. coli*, as described in Figure 4 and Materials and Methods, using donor plasmid TeI4c/4c, which expresses the TeI4c-ΔORF intron and TeI4c IEP (2nd ATG), and recipient plasmids that contain target sites for the TeI4a, b, c, d, and e introns (i.e., ligated E1-E2 sequences flanking the intron-insertions sites in *T. elongatus*). Donor plasmid expression was induced with 500 μM IPTG for 1 h at 48 °C. Mobility efficiencies were calculated as the ratio of (Tet^R + Amp^R)/Amp^R colonies and are the mean of two independent experiments. The two repeats for the TeI4b target site gave mobility efficiencies of 83% and 72%, and the variation between repeats for the other target sites was less than 3-fold. Similar results were obtained with the Te4Ic IEP expressed from the first ATG (not shown). Insertion of the intron at the expected target site was confirmed by PCR (12 colonies) and sequencing (3 colonies) in all cases, except for the TeI4d and TeI4e target sites, where 100% and 60%, respectively, of the colonies did not give the expected PCR product and thus may not correspond to be bona fide mobility events. In a related experiment, donor plasmid TeI3c/4c, which expresses the TeI3c intron and TeI4c IEP, likewise gave high mobility efficiencies (60%–80%) with alternative target sites (unpublished data).
